# Hierarchically Structured and Tunable Hydrogel Patches: Design, Characterization, and Application

**DOI:** 10.1002/smll.202407311

**Published:** 2024-11-20

**Authors:** Lea Steinbeck, Richard Paul, Julia Litke, Isabel Karkoszka, G. Philip Wiese, John Linkhorst, Laura De Laporte, Matthias Wessling

**Affiliations:** ^1^ Chemical Process Engineering AVT.CVT RWTH Aachen University Forckenbeckstraße 51 52074 Aachen Germany; ^2^ DWI – Leibniz‐Institute for Interactive Materials e. V. Forckenbeckstraße 50 52074 Aachen Germany; ^3^ Institute of Technical and Macromolecular Chemistry RWTH Aachen University Worringerweg 1‐2 52074 Aachen Germany; ^4^ Center for Biohybrid Medical Systems (CBMS) Advanced Materials for Biomedicine (AMB) Institute of Applied Medical Engineering (AME) University Hospital RWTH Aachen Forckenbeckstraße 55 52074 Aachen Germany; ^5^ Present address: Process Engineering of Electrochemical Systems Technical University of Darmstadt Otto‐Berndt‐Str. 2 64287 Darmstadt Germany

**Keywords:** biomaterial, hierarchical cellular material, hydrogel, porosity, projection lithography

## Abstract

Recent studies show the importance of hydrogel geometry for various applications, such as encoding, micromachines, or tissue engineering. However, fabricating hydrogel structures with micrometer‐sized features, advanced geometry, and precise control of porosity remains challenging. This work presents hierarchically structured hydrogels, so‐called hydrogel patches, with internally deviating regions on a micron‐scale. These regions are defined in a one‐step, high‐throughput fabrication process via stop‐flow lithography. Between the specified projection pattern during fabrication, an interconnecting lower crosslinked and more porous hydrogel network forms, resulting in at least two degrees of crosslinking within the patches. A detailed investigation of patch formation is performed for two material systems and pattern variations, revealing basic principles for reliable patch formation. In addition to the two defined crosslinked regions, further regions are implemented in the patches by adapting the pattern accordingly. The variations in pattern geometry impact the mechanical characteristics of the hydrogel patches, which display pattern‐dependent compression behavior due to predefined compression points. Cell culture on patches, as one possible application, reveals that the patch pattern determines the cell area of L929 mouse fibroblasts. These results introduce hierarchically structured hydrogel patches as a promising and versatile platform system with high customizability.

## Graphical abstract

This work presents hierarchically structured hydrogel networks, so‐called hydrogel patches, with internally deviating highly crosslinked (HC) and low crosslinked (LC) regions on a micron‐scale. These regions are entirely tailorable and pre‐defined in a one‐step, high‐throughput fabrication. Thus, their characteristics, e.g. their porosity or their mechanical characteristics, are adjustable, introducing the patches as promising and versatile platform systems.

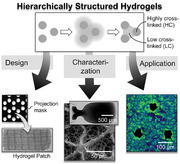

## 1 Introduction

Hydrogels are a group of functional materials that consist of crosslinked polymer networks and that drastically swell in water or other solvents. With their beneficial attributes such as their tunable size, porous network, responsiveness to external stimuli, and biocompatibility, they show great potential to meet challenges in optics, separation and transport processes, and tissue engineering, among others.^[^ ^1^, ^2^, ^3^, ^4^, ^5^, ^6^, ^7^ ^]^ To provide a high level of complexity within these functional materials, not only the material itself but also the structure of the hydrogels is essential.^[^ ^2^, ^4^ ^]^ Recent studies show the benefits of an anisometric geometry of hydrogels, like an improved diffusion efficiency or an increased attachment area due to the increased surface‐area‐to‐volume ratio of anisometric hydrogels.^[^ ^5^, ^8^ ^]^ When the hydrogel geometry is designed, structure‐dependent applications such as encryption units, micromotors, or cell guidance become possible. Complex hydrogel structures further enable a more suitable adaption of the hydrogels to their desired purposes and, thus, more successful applications in general.^[^ ^5^, ^8^, ^9^, ^10^ ^]^

Especially for biomedical applications, highly structured hydrogels are of great relevance, as they can, for example, significantly influence cell interactions, migration, tissue shape, and drug delivery.^[^ ^5^, ^10^, ^11^, ^12^, ^13^ ^]^ The hydrogels then establish the microenvironment for cells during these applications. In turn, the cell behavior directly relates to the mechanical and topographical characteristics of the surrounding microenvironment. It is desirable to engineer the characteristics of the hydrogels to the natural niche of the cell type of interest.^[^ ^11^, ^14^, ^15^ ^]^ Mechanical characteristics comprise, for example, the stiffness of the hydrogel scaffolds, which can change the cells' morphology, mobility, proliferation, or gene expression, altering cell differentiation.^[^ ^16^ ^]^ The stiffness of a hydrogel polymer network is related to its crosslinking degree and its pore characteristics, i.e. pore size, degree of porosity, heterogeneity, and 2D and 3D topology. A versatile process to control such features all at the same time is also desirable to meet the needs of biomedical applications. Pore size and degree of porosity additionally influence the diffusion of nutrients and other essential supplies to cells and the removal of metabolic products, ensuring the sustained maintenance of the cells.^[^ ^14^, ^17^ ^]^ In addition to the stiffness, adherent cells require a substrate of suitable surface topography and macroporosity, which enables cell adhesion and in‐growth similar to the natural extracellular matrix. Therefore, void spaces between hydrogel structures in the size of the cells and larger are essential for cell infiltration. The shape and size of these void spaces significantly influence the cells' behavior.^[^ ^4^, ^9^, ^18^ ^]^ Beyond that, the topography can stimulate the cells and instruct cellular reactions and cell fate.^[^ ^19^ ^]^ In larger architectures, the topography helps to recreate the physiological complexity of the microenvironment of the cells and to mimic natural structures such as organs.^[^ ^20^, ^21^ ^]^ Hence, for all hydrogel applications, particularly in biomedicine, it is essential to precisely define the crosslinking degree on the molecular level as well as the architecture of individual hydrogels or their assemblies in the micrometer range.

In recent years, various methods have been developed and established to meet these structural challenges. The probably most widely used approach to achieve structured hydrogels is the microporous annealed particle (MAP) assembly.^[^ ^22^, ^23^, ^24^, ^25^ ^]^ Thereby, building blocks are crosslinked, which are often randomly arranged spherical microgels.^[^ ^4^ ^]^ However, systems exist where the arrangement is not random but, for example, controlled via aspiration of single spherical building blocks, resulting in highly structured layers.^[^ ^26^ ^]^ Although the packing of MAP scaffolds built from spherical microgels can be adjusted to a certain degree by adapting the microgels' arrangement or diameter, the respective assemblies are still limited in pore shape.^[^ ^4^, ^27^ ^]^ A variation in pore shape and interconnectivity is achieved by using non‐spherical building blocks.^[^ ^9^, ^18^ ^]^ Compared to ordinary spherical microgels, these anisometric building blocks offer several advantages, namely higher surface area, higher diffusion efficiency, and increasing porosity within assemblies.^[^ ^4^, ^5^, ^14^ ^]^ Anisometric building blocks with additional features like being magnetically responsive,^[^ ^28^, ^29^ ^]^ biphasic,^[^ ^30^ ^]^ or amphiphilic^[^ ^31^ ^]^ can be arranged in a partially controlled manner, resulting in an induced directionality, more homogeneous pore spaces, or more defined arrangements. Furthermore, a controlled stacking of single anisometric, complex‐shaped building blocks can result in a structurally ordered assembly.^[^ ^32^ ^]^ Other approaches to achieving structured hydrogels are not based on assemblies of building blocks but rather on the patterning of larger hydrogel sheets by molding to achieve pockets,^[^ ^10^ ^]^ geometrical patterns,^[^ ^19^ ^]^ curvatures,^[^ ^33^ ^]^ or grooves.^[^ ^34^ ^]^ Approaches with probably the most control and shape variety of hydrogels on a micron scale are based on lithography, enabling the creation of complex, coherent structures.^[^ ^35^, ^36^ ^]^ These structures often display cellular structural elements as one of eight bioinspired structural design elements defined in the literature.^[^ ^37^ ^]^ However, these structures comprise only one degree of crosslinking within the polymer network separated by void spaces and lack gradients in the polymer. Combining cellular and gradient structural elements by providing structured networks with defined regions of deviating crosslinking degrees would result in an even increased number of applications or an enhanced realization of existing applications. Locally resolved crosslinking, for example, can initiate cell migration and proliferation to intended regions of the cells' preferred stiffness.^[^ ^38^ ^]^ By controlling the topography of these regions, multicellular tissues such as co‐cultures could be designed, which offer close‐to in vivo conditions compared to existing approaches.^[^ ^39^ ^]^ Human umbilical vein endothelial cells (HUVEC) and normal human dermal fibroblasts (NHDF) comprise such a promising co‐culture to investigate vascularization on intricate durotactic and topotactic scaffolds. Furthermore, structured networks with defined crosslinking regions can help to understand multicellular processes or even single‐cell behavior by representing a tunable platform system and thereby precisely tuning the cells' environment.^[^ ^38^ ^]^ This understanding could include finding cell type specific optimal substrate properties or screening the efficacy of drugs, among others.^[^ ^40^ ^]^ Beyond that, non‐biomedical applications would also benefit from hydrogels with patterned crosslinking. In soft robotics, for example, the degree of crosslinking can be used to control the swelling and deswelling of hydrogels. Being able to design crosslinking patterns within a hydrogel can result in a controlled motion, such as advanced folding patterns.^[^ ^41^ ^]^

This work presents the fabrication and characterization of hierarchically structured hydrogel patches by stop‐flow lithography (SFL), comprising cellular and gradient structural design elements. A transparency mask shapes UV light that initiates localized photopolymerization within a prepolymer‐containing microfluidic channel. The features of the sheetlike hydrogels form by locally exceeding a particular input energy, resulting in at least two regions with variable crosslinking degrees. The internal structure and the crosslinking degrees of the patches can be adjusted. Hence, the presented fabrication method enables the fabrication of precisely structured hydrogels across many length scales. The patch formation is investigated for varied exposure parameters for two material systems based on poly(ethylene glycol) diacrylate (PEGDA) and *N*‐isopropyl acrylamide (NIPAM). Additionally, the impact of mask parameters, such as spot diameter and distance, are studied for the NIPAM‐based material system to determine reliable patch formation parameters. The porous structures of poly NIPAM (PNIPAM) patches with varying crosslinking patterns and degrees are visualized by field emission scanning electron microscopy (FESEM) after freeze‐drying. Microfluidic compression and squeezing experiments are performed to characterize the impact of the patch patterns on their mechanical properties. Finally, since tissue engineering is a promising application for hydrogel patches, their usage as cell scaffolds is showcased for cultivating L929 mouse fibroblast cells in microtiter plate experiments.

## 2 Results and Discussion

### 2.1 Hydrogel Patch Formation

**Figure 1:**
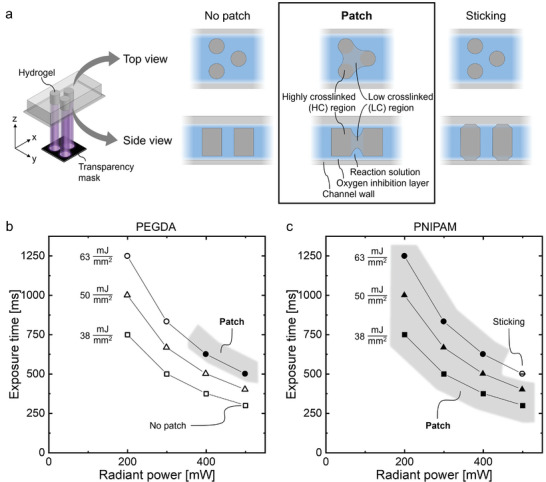
Hydrogel formation using mask projection lithography. a) Schematics of the hydrogel formation events in the side and top view for a so‐called hexagonal spot arrangement, showing the network connections. b,c) Occurrence of the formation events depending on the applied exposure parameters during fabrication, displayed for three radiant exposures (38 mJ/mm^2^  (square), 50 mJ/mm^2^  (triangle), and 63 mJ/mm^2^  (circle)) with four parameter combinations (radiant exposure | exposure time) each. The occurring formation events for a (b) PEGDA‐based and a (c) PNIPAM‐based material system are displayed at these exposure parameters equal for both systems: *no patch* (unfilled symbols), *patch* formation (filled symbols and a gray area), and *sticking* (strikethrough symbols). A transparency mask with circle‐shaped spots of 40 µm diameter was used with the circles having an edge‐to‐edge distance of 100 µm to all six neighboring circle shapes on the mask (hexagonal arrangement).

Hydrogel patches are hierarchically structured hydrogel networks consisting of highly crosslinked (HC) and low crosslinked (LC) regions. The locations of the HC regions of the patches are defined by a transparency mask in the projection lithography fabrication process, inducing a localized polymerization within the reaction solution. The LC regions within the patches arise from light scattering and radical diffusion that is achieved by irradiating in a specific parameter range, depending on the transparency mask pattern. Within this range, the polymerization is not limited to the defined irradiation spots but additionally occurs to a lesser extent in the intermediate surroundings, where the reduced local energy leads to decreased crosslinking and the formation of the corresponding LC regions. Thus, a so‐called hydrogel patch arises, consisting of an HC pattern defined by the transparency mask and connected by an LC polymer network. The patches investigated in this work are ≈2.25 mm long, 0.9 mm wide, and 80 µm high, which is defined by the dimensions of the microfluidic channel used for fabrication and the irradiation area of the projection lithography setup. By adapting these dimensions, the overall patch size can be varied. Using the stop‐flow mode of the projection lithography setup provides a high‐throughput fabrication (> 50 min^−1^) of the patches. **Figure** **1**a shows a visualization of the top and side view of a patch inside a microfluidic channel. The HC regions defined by the mask are visualized in dark gray, whereas the LC regions are light gray. Besides *patch* formation, other formation events can occur, namely, *no patch* or *sticking*. *No patch* means the formation of hydrogels with only one degree of crosslinking or no hydrogel network at all. These hydrogels, which are simply referred to as uniformly crosslinked, correspond to the given pattern of the mask and are separated from each other without having an LC interconnection and thus without forming a patch (compare Figure 1a *no patch*). During *sticking*, the arising hydrogel network sticks to the channel bottom, top, or both (compare Figure 1a *sticking*). Hence, the hydrogel cannot be flushed out of the microfluidic channel and collected. Instead, the hydrogel blocks the channel. The *sticking* can occur with uniformly crosslinked hydrogels or with patches and leads in both scenarios to the interruption of the continuous fabrication process. However, in applications of microfluidic chromatography, this may actually be desirable to fabricate a precisely tuned stationary phase of the chromatography bed. Further distinctions of the presented formation events and their origin are discussed in Section S2.1 (Supporting Information). For both formation events, *no patch* and *sticking*, no patches can be collected for subsequent use. Hence, the first objective of the study was to identify at which conditions patches form.

Which formation event occurs depends on several influencing parameters. The hydrogel formation via free‐radical photopolymerization is mainly determined by irradiation, polymerization system, mass transport by diffusion, light scattering, and oxygen diffusion.^[^ ^42^ ^]^ The irradiation parameters determine the initiation of the polymerization, whereas the molecular composition of the polymerization system determines the reactivity of the reaction solution. Monomer diffusion and light scattering cause the diffusion of the local reactivity resulting in less sharp polymerization domains. This apparent disadvantage was utilized to generate hydrogel patches. Oxygen prevents radical photopolymerization, and oxygen needs to be depleted before crosslinking reactions can occur. At the channel walls of the microfluidic flow channel, a so‐called oxygen inhibition layer (OIL) is present since atmospheric oxygen diffuses through the porous channel material poly(dimethylsiloxane) (PDMS). Thus, the polymerization at the channel walls is prevented to a certain extent, and the thickness of the OIL influences the height and the sticking of the hydrogels.^[^ ^42^, ^43^ ^]^ Radiant power directly correlates with the number of radicals forming,^[^ ^44^ ^]^ and the exposure time highly impacts the diffusion since this is time‐dependent. As a result, hydrogels are shorter (z‐axis) and thicker (xy‐plane) when fabricated with decreased radiant power and increased exposure time for constant energy input. This hydrogel shape varies from the straight elongated hydrogel shape normally desired during SFL.^[^ ^42^ ^]^ However, to achieve 3D patches, these shape variations toward shortened and thickened hydrogels are favorable since they prevent sticking by an increased distance to the channel walls and broaden the hydrogel network, particularly in its height center. Hence, a parameter combination with low radiant power and high exposure time is preferred for successful patch formation at a constant energy input. Generally speaking, a decreased energy input results in *no patch* formation, whereas an increased energy input favors *sticking*. Furthermore, for successful patch formation, precursor monomer diffusion and light scattering need to be enhanced compared to *no patch* formation, whereas the oxygen inhibition at the channel walls needs to be retained. Thus, the LC hydrogel network can form beyond the irradiated areas and connect individual HC regions without *sticking* to the channel wall. To achieve mechanically stable and removable patches, the parameters of the fabrication process need to be controlled and set within a certain interval.

The most prominent process parameters for controlling formation events are the material system, the exposure parameters, and the mask dimension. These parameters were tuned within this study to form hydrogel patches successfully and reliably. Figure 1b,c display the formation events for two different material systems and varying exposure parameters. The first material system is based on poly(ethylene glycol) diacrylate (PEGDA), whereas the other material system contains *N*‐isopropyl acrylamide (NIPAM) and *N*, *N*'‐methylene bisacrylamide (BIS). A transparency mask was used that has circle‐shaped spots of 40 µm diameter, which are arranged in a hexagonal arrangement with 100 µm edge‐to‐edge distances to all six neighboring circle shapes on the mask. The exposure time *t* and the radiant power *P* of the UV‐LED directly correlate with the energy input on the irradiation area *A* according to $H=\frac{P\cdot t}{A}$.

Three different values of this energy input per area, called radiant exposure *H*, were studied: 38, 50, and 63 mJ/mm^2^. For each radiant exposure, the exposure time and the radiant power were varied, investigating a total of four parameter combinations per radiant exposure, resulting in a total of 12 investigation points. The formation events, *patch*, *no patch*, or *sticking*, were recorded for both material systems.

Figure 1b shows the formation events occurring for the PEGDA material system, displaying exposure time over radiant power while grouping the parameter combinations displaying the same radiant exposure. *Patch* formation occurred at the highest radiant exposure of 63 mJ/mm^2^  applied for the two parameter combinations with the highest radiant powers but lowest exposure times (500 mW for 500 ms, 400 mW for 625 ms). For all other parameter combinations, *no patches* were fabricated, either forming hydrogels without LC interconnection or showing no hydrogel network at all. Hence, the HC regions could not be connected for radiant exposures below 63 mJ/mm^2^  for the PEGDA material system. Patches forming only at high radiant power and low exposure times for a constant radiant exposure first is counter‐intuitive. For high radiant powers and low exposure times, the HC regions at the irradiation spots are theoretically thinner (xy‐plane) and longer (z‐axis). However, the other two combinations of parameters of the same radiant exposure with thicker (xy‐plane) and shorter (z‐axis) HC regions did not result in *patch* formation. Thus, the hydrogel network between the HC regions seemed not to be sufficient for a stable polymer network. A possible explanation could be the height confinement of this network in the center for low radiant powers and high exposure time, possibly being too thin to withstand mechanical stress.

For the NIPAM‐based material system, a wider formation window was observed for the same exposure parameters, as shown in Figure 1c. At the highest radiant exposure of 63 mJ/mm^2^ , the parameter combination with the highest radiant power (500 mW, 500 ms) resulted in *sticking* of the resulting hydrogel network. For this parameter combination, *sticking* is comparably favored due to a higher (z‐axis) hydrogel network for high radiant powers. Except for this single‐parameter combination, *patch* formation was achieved for all applied parameters.

The results show a significant influence of the material system on the formation of hydrogel patches. For the same weight proportion of PEGDA and NIPAM, *patch* formation is significantly more favored for the NIPAM‐based material system. In this context, the total number of reactive acrylate groups in the reaction solution is decisive for the hydrogel and thus the *patch* formation.^[^ ^15^ ^]^ To calculate this number, the number of acrylate groups per molecule, as well as the molecule's mass, are relevant since the reaction solutions were composed via weight proportions. Furthermore, the NIPAM‐based material system not only consists of NIPAM as the monomer but also includes the crosslinker BIS, whose reactive groups must also be considered. The NIPAM‐based material system, therefore, has almost three times as many acrylate groups as the PEGDA‐based material system. Moreover, there are further influencing factors regarding the hydrogel formation that differ between both material systems, such as rheological properties (compare Section S2.2, Supporting Information), molecular diffusion, or accessibility of reactive acrylate groups. The molecular diffusion is directly affected by the coil volume of the molecules.^[^ ^15^ ^]^ Since the polymer‐forming molecules of the NIPAM‐based material system are both significantly smaller than the PEGDA molecules, their molecular diffusion is faster. Hence, the favored patch formation of the NIPAM‐based material system is reasonable due to the increased acrylate groups and the faster molecular diffusion of the NIPAM‐based material system. Due to the favored *patch* formation of the NIPAM‐based material system for the same weight proportion of the precursor and the thermo‐responsive properties of PNIPAM with arising application possibilities, all the following investigations in this work are conducted with PNIPAM patches.

**Figure 2:**
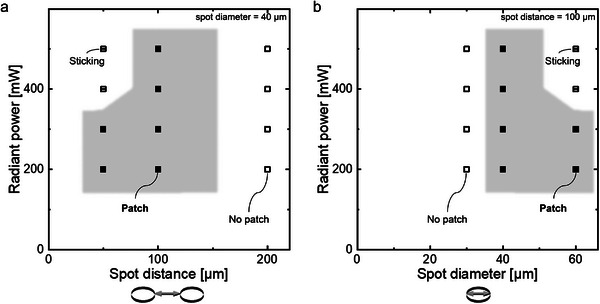
Influence of the dimensions of a transparency mask with a hexagonal arrangement of circle‐shaped spots on the hydrogel formation events: *no patch* (unfilled symbols), *patch* formation (filled symbols and a gray area), and *sticking* (strikethrough symbols). PNIPAM hydrogels fabricated at a constant radiant exposure (38 mJ/mm^2^ ), investigated for a) a varying spot distance at a constant spot diameter of 40 µm and b) a varying spot diameter at a constant spot distance of 100 µm for four exposure combinations each.

**Figure 3:**
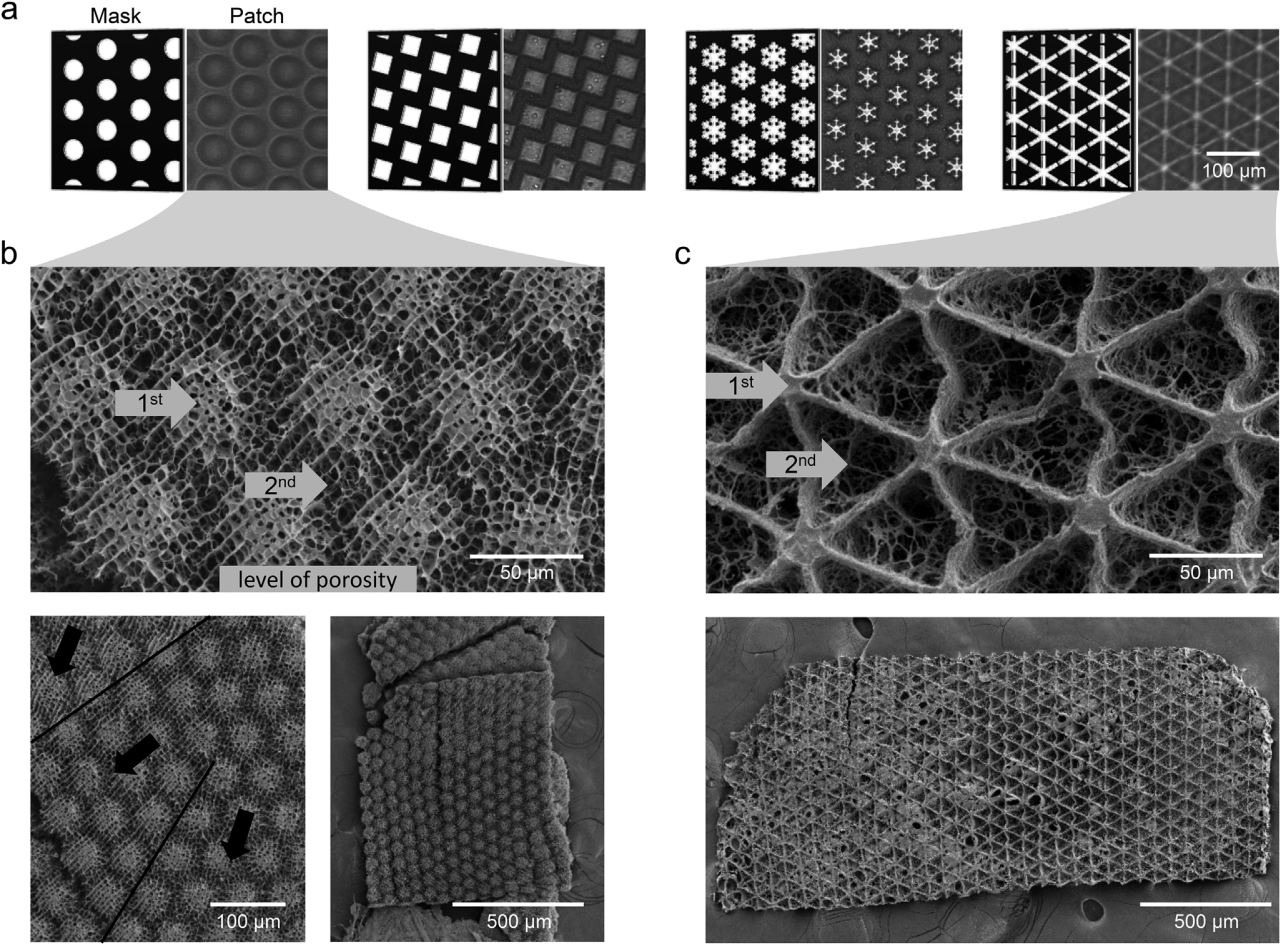
Varying patch geometries by different spot shapes. a) Mask schemes and corresponding brightfield images of patches with different shapes of the HC regions induced by the irradiated mask spots. From left: circle‐shaped, square‐shaped, snowflake‐shaped, star‐shaped. All other mask parameters are held constant for all patch types: irradiation spot area, center‐to‐center spot distance, and hexagonal spot arrangement. Scale bar (100 µm) applies to all micrographs. b,c) Field emission scanning electron microscopy (FESEM) images of patches with (b) circle‐shaped or (c) star‐shaped irradiation spots. (b) Black lines and arrows indicate regions with different directionalities within the porous structure of the patch, most likely resulting from freeze‐drying.

Besides the material system and the exposure parameters, the features of the transparency mask, i.e. the geometrical details and their dimensions, highly influence whether patches are formed and further define the geometry of the patches. The geometrical features of the mask comprise the shape of the single irradiation spots, their diameter, their distance from each other, and their lateral arrangement on the mask. **Figure** **2** shows the formation events for varying the spots' a) distances and b) diameters. During the variation of one of these mask parameters, the other parameter was held constant at 40 µm diameter or 100 µm distance, respectively. All masks have circle‐shaped irradiation spots in a hexagonal arrangement, which is exemplified in Figure 1a (and **Figure** **3**a). Each spot has six directly neighboring spots, separated by the same distance. During fabrication, the mask pattern is projected into the microfluidic channel, reducing its dimensions by a constant factor of 5.46 as a result of the projection lithography setup.^[^ ^45^ ^]^ Therefore, the resulting patches fabricated with a mask with a spot diameter of 40 µm exhibit HC regions with diameters of ≈7 µm. To investigate the influence of the mask on the patch formation, the formation events for varying mask dimensions were investigated for PNIPAM hydrogels at constant radiant exposure (38 mJ/mm^2^ ), but radiant power and corresponding exposure time were varied, equal to the parameter combinations investigated before (compare Figure 1c). If the diameter of the irradiation spots on the transparency mask was held constant at 40 µm, an ideal spot distance of 100 µm was observed (compare Figure 2a). For this spot distance, each applied parameter combination results in *patch* formation. For a halved spot distance of 50 µm, in only two of the four irradiation cases, patches were formed. A decreased spot distance enhances *sticking* of the hydrogels due to the mask pattern‐dependent increased local energy input and the decreased diffusion and scattering distances, resulting in lower oxygen inhibition. Furthermore, high radiant power at constant radiant exposure favors *sticking* as a result of increased oxygen depletion at the channel walls. The local energy input can be described by the proportion of the actual irradiated area, relative to the possible overall irradiation area. This value equals the proportion of HC regions in relation to the patch area. The mask with a decreased edge‐to‐edge spot distance of 50 µm leads to a proportion of 18% of the actual irradiated area. In comparison, the reference mask with 100 µm spot distance and the same spot diameter has an actual irradiation contribution of 7% (compare Table S1, Supporting Information). For a doubled spot distance of 200 µm, *no patch* formation occurs for all parameter combinations. Here, the same effects cause this formation event, which work as the opposite by decreasing the local energy input (3% actual irradiated area) and increasing the diffusion and scattering distances with increasing spot distance.

Not only does a decreasing spot distance increase the local energy input per area, but an increasing spot diameter for a constant spot distance has the same effect. Figure 2b shows the formation events for a varying spot diameter and the four defined parameter combinations at 38 mJ/mm^2^  radiant exposure each. If the spot diameter is increased only by 50% to 60 µm, *sticking* occurs for the two‐parameter combinations with the highest radiant powers (400 mW, 500 mW). Here, the actual irradiated area accounts for 13% of the possible overall irradiation area compared to 7% of the reference mask with 40 µm diameter (compare Table S1, Supporting Information). However, in contrast to the varying spot distances, the diffusion and scattering distances between the irradiation spots stay the same for varying spot diameters due to a constant edge‐to‐edge distance. Hence, the differences in formation events are only caused by the deviating local energy inputs due to adapted actual irradiation areas. In contrast, *no patch* appears for a spot diameter of 30 µm for all combinations of exposure parameters tested. For this mask, a decreased actual area of 5% is irradiated, resulting in an insufficient local energy input for *patch* formation.

Investigating the influence of the dimensions of the transparency mask enables reliable hydrogel patch fabrication. As a reference point, the spot diameter‐to‐distance ratio should be 0.4 or slightly greater to definitely achieve patches during fabrication. A lower ratio results in *no patch* formation and a significantly higher ratio in *sticking*. Based on this ratio, the geometry of the mask can be varied, revealing straightforward patch formation, as discussed in the following section. Even when there is an optimal ratio of mask dimensions to form patches, these dimensions can be adjusted to a certain extent. This flexibility can be further enhanced by additionally adapting the fabrication parameters. Thus, the extent of the LC network and the ratio between the LC and HC networks are tunable. This, in fact, allows the design of the patch dimensions on purpose and tailored to applications, for example, a cell‐specific space for growing. To allow comparability between the patches characterized in this work, all the following PNIPAM patches were fabricated at the same exposure parameter settings (38 mJ/mm^2^ , 200 mW, 750 ms).

### 2.2 Geometry and Porosity Variation

With the spot diameter‐to‐distance ratio of ⩾ 0.4, it is straightforward to design new patch geometries. On the one hand, the HC spots can be significantly enlarged, as exemplarily shown in Figure 3a for a patch with enlarged circles. This patch compromises 52 µm large circles in contrast to the diameter of the circles of the previously investigated patches of 7 µm. The enlargement was directly realized by increasing the diameter and distance of the spots while retaining the approximate diameter‐to‐distance ratio. On the other hand, the shape of the irradiated spots and, thus, the shape of the HC regions can be tailored as well. Figure 3a shows four different masks and their resulting patches with adapted spot shapes. For all masks, the spots' surface area, their distance from center to center, and their hexagonal arrangement are identical.

Field emission scanning electron microscopy (FESEM) was performed to reveal the polymeric structure of the different hydrogel patches. Figure 3b,c shows the polymeric structures of patches with a circle‐shaped and a star‐shaped geometry, respectively. Less and more porous regions are present within the patches, corresponding to the patch‐specific HC and LC regions observed before. The HC regions induced by the spots of the mask are comparatively denser than the interconnecting LC regions. Thus, the irradiation spot location is confirmed to be directly related to a higher degree of crosslinking, as stated before, expressed by a lower porosity in electron microscope images. These two regions within the patches represent two levels of porosity that can be intentionally designed with the fabrication method presented here. The first level refers to the spot‐induced HC regions, while the second level of porosity evolves from the bridging of the polymer network between these spots (compare Figure 3b,c).

The apparent directionality within the polymeric structure of the circle‐shaped patch (compare Figure 3b) is most likely related to the preparation method of the patch sample and, therefore, not to the structural features of the patch itself. The chosen drying method during sample preparation highly affects the appearance of hydrogel structures in FESEM investigations.^[^ ^46^ ^]^ Freeze‐drying used here applies a force on the samples and is known to introduce pores within soft polymeric structures, and the direction of ice crystal growth during freeze‐drying mostly likely induced the directionality within the patch. This effect and comparable polymeric structures are known in the literature for freeze‐dried hydrogels. When drying the hydrogel with other methods, no directionality within the polymeric structure is observed, supporting the given hypothesis that the freeze‐drying process is responsible for this directional appearance and it is no feature of the patches themselves.^[^ ^46^, ^47^ ^]^ Furthermore, a cause related to the fabrication is unlikely since a possible flow‐ or light‐induced cause would result in a unidirectional impact on the patch structure, whereas the apparent directionality changes within the patch, indicated by the black lines and arrows in the middle magnification of Figure 3b.

The height (z‐axis) of the hydrogel differs between regions with low and high crosslinking or porosity. This height difference is particularly visible for the FESEM image of the star‐shaped patch (compare Figure 3c). The HC regions appear particularly dense and possess the approximate height of the channel in which the patches were fabricated. In contrast, the LC regions between exhibit µm‐sized pores after freeze–drying and seem like triangle‐shaped cavities due to their comparatively low height. Since the LC regions receive less energy during formation compared to the HC regions, they exhibit a comparatively lower height. In the photo‐induced radical polymerization of the patches using projection lithography, the highest energy is concentrated at the focal height of the light in the center of the microfluidic channel. At this level, the radiant exposure is maximized due to the light focus, and the oxygen inhibition is minimized due to the greater distance from the channel walls.^[^ ^43^ ^]^ Consequently, the formation of LC regions between the irradiation spots starts at the z‐axis centered height of the channel surrounding the irradiation spots (in the xy‐plane). As the distance to the channel walls decreases, oxygen inhibition increases, necessitating high local energy at the top and bottom of the channel for polymerization, which the LC regions lack. Thus, both crosslinked regions develop in the central height of the formed patches, though only the HC regions span the full channel height, extending in both directions along the z‐axis (compare Figure 1a *patch* side view).

**Figure 4:**
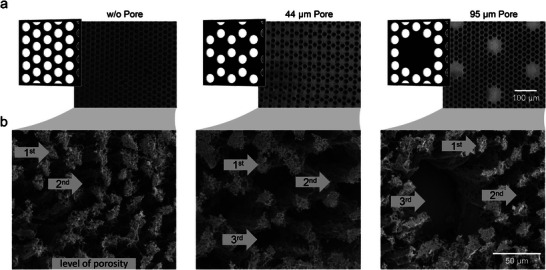
Patches series with induced third level of porosity by missing out irradiation spots within a regular hexagonal spot arrangement of circle‐shaped spots with 7 µm diameter on the patches. From left to right: without additional induced porosity, induced 44 µm porous regions by single missing irradiation spots, induced 95 µm pores by missing an arrangement of seven spots. Visualization of the patches by a) mask schemes and corresponding brightfield images of the patches and b) FESEM images with levels of porosity being indicated. Scale bars (a: 100 µm, b: 50 µm) apply to all micrographs each.

In addition to the two induced and always occurring degrees of crosslinking of the patches, further degrees can be induced, being equivalent to levels of porosity. **Figure** **4** shows two exemplarily patches with three engineered levels of porosity (Figure 4 44 µm *pore* and 95 µm *pore*), based on mask designs having larger domains without light transmission. This third level of porosity is introduced by varying the distance between some irradiation spots within the patch while maintaining the hexagonal spot arrangement with optimal diameter‐to‐distance ratio at other regions within the mask. Thus, the formation of coherent patches is ensured by retaining the basic framework of spots that was designed for reliable patch formation in Section 2.1. The three exemplary patch structures in Figure 4 have the same first two degrees of crosslinking with HC and LC regions, resulting in two porosity levels. All three patches have circle‐shaped HC regions with 7 µm diameter and 18 µm edge‐to‐edge distance to each other as the first level of porosity. In between, LC regions are present, possessing the second level of porosity. By omitting single irradiation spots within the mask, the second patch has regions with an even lower crosslinked network and, thus, a third level of porosity, which is highlighted in the FESEM images in Figure 4b 44 µm *pore*. These regions cover a diameter of 44 µm and consist of a polymeric network, which is visible during brightfield imaging but cannot be reliably detected in the FESEM images, which is most probably attributable to the preparation method for FESEM via freeze–drying. Within the mask used to fabricate the third patch (Figure 4 95 µm *pore*), not only single spots but entire spot arrangements of seven spots are omitted. Hence, porous regions with a diameter of 95 µm emerge within the resulting patch. Since this distance between the irradiation spots is too large to be bridged by the forming polymer network during the fabrication, these porous regions actually are single pores, respectively, through‐pores within this patch type. By omitting different irradiation spots within the mask pattern, a customizable third‐level porosity is induced into the patch pattern.

The presented patch examples demonstrate the design freedom allowing to potentially adjust the patch properties beyond the previously described combinations of HC and LC regions. Consequently, other regions within the patch pattern can be adapted, enabling a high degree of freedom and multiple levels of porosity even beyond the three shown. The variable spot shape and the intended dark spots of the irradiation pattern of the transparency mask increase the complexity of the geometry and also allow the design of laterally inhomogeneous porosity patterns. Such designed patch patterns may, for instance, provide tissue scaffold structures, which enable a directionality and cell growth guidance structure, or tune the mechanic properties of the patch at desire. Introduced through‐pores can further serve, for example, as migration channels of cells for applications of the patches in cell culture. They also allow the stacking of patches while having connection channels and, thus, provide thicker scaffold structures.

### 2.3 Designed Mechanical Characteristics

**Figure 5:**
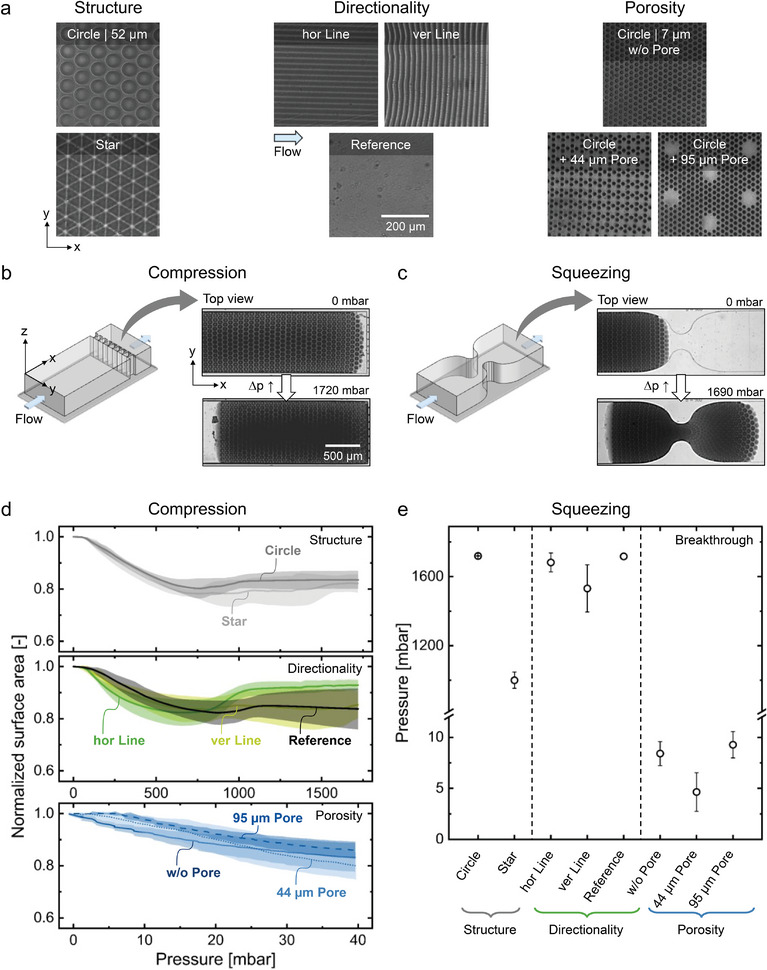
Mechanical properties of selected patches. a) Investigated patches (top view) grouped according to geometrical variations: structure, directionality, and porosity. Scale bar (200 µm) applies to all micrographs. b,c) Schematic microfluidic channel geometry and experimental process of (b) lateral compression with the compressive force being applied along with the flow direction and (c) squeezing experiments, exemplarily shown for a patch with circle‐shaped HC regions with 52 µm diameter in a hexagonal arrangement (*circle* | 52 µm). Scale bar (500 µm) applies to all micrographs. d) Compression behavior of the patches, displayed by the change in their surface area over increasing applied pressure. e) Squeezing behavior of the patches, characterized by the pressure needed for the patches to break through the channel constriction.

The crosslinking degree and the patterns and combinations of the levels of porosity of a patch can influence its mechanical characteristics. Due to the pattern of HC and LC regions, local mechanical differences occur within the patch laterally. The overall mechanical characteristic of each patch type, meanwhile, results from an interplay of these regions, which is determined by the geometrical pattern of the masks. To illustrate this feature of the patches, selected patches, which are shown in **Figure** **5**a, are investigated regarding their mechanical characteristics. The patches are classified according to their geometry and the associated influence on their mechanical properties (compare Table S1, Supporting Information, for geometric parameters of the patches). By comparing these patches according to their classifications, the influence of the individual aspects of the patches' patterns on their mechanical characteristics is investigated. The mechanical characteristics of the patches were determined by performing lateral compression and squeezing experiments inside microfluidic channels. The channels have the yz‐dimensions of the patches and are schematically displayed in Figure 5b,c. The microfluidic channel used for the compression experiments has a filter structure at the end of the channel, which restricts the patches and lets the surrounding water permeate. Hence, the patches were pressed against the filter, as shown in Figure 5b for the *circle* patch. Micrographs of the uncompressed, the maximal compressed, and the end state of each patch type are displayed in Figures S6–S8 (Supporting Information). For the quantitative investigation of the patches' compression ability, the area visible in the top view was measured as a function of the applied pressure. Since a linear pressure ramp was applied, the pressure change is proportional to time. The change of the normalized area over applied pressure is visualized in Figure 5d, showing the averaged curve progression of the eight selected patch types. Monitoring the patches' surface area during compression reveals the compression ability of the patches depending on their geometrical pattern.

The analysis of the compression curves allows to categorize the behavior of patches into three distinct compression regimes: linear, plateau, and reverse compression. According to the linear regime, the area of the patch decreases linearly with increasing pressure, like the curve of the *w/o pore* patch. During the plateau regime, the patch area linearly decreases to a certain minimum surface area before staying constant, similar to the curve of the *circle* patch. The reverse‐compression regime is characterized by an initial strong decrease in patch area over applied pressure followed by a reversal, as visible for the curve of the *hor line* patch. This unexpected behavior is attributed to changes in force distribution caused by the patch blocking the filter structure of the microfluidic channel. Since the HC regions are denser than the LC regions, they block the microfluidic channel to a greater extent. Thus, patches with a great proportion of HC regions preferably exhibit this compression behavior. See Section S5 (Supporting Information), for a detailed discussion on the patches' compression behavior. The three distinct compression regimes classify the patches' compression behavior, and the specific regime observed is influenced by various porosity features of the patches' geometrical patterns. The method and results described above require a detailed simulation study to correlate porosity features with quantitative mechanical behavior. However, this is beyond the scope of this contribution.

The structure of HC regions of the patches significantly affects their compression behavior. *Star* patches all compress according to the reverse compression regime, whereas only some *circle* patches show a reverse compression and the others a plateau compression. On average, the *star* patch type compresses more than the *circle* patch type, despite having the same proportion of HC and LC regions. This difference is likely due to the thinner HC regions in *star* patches. Additionally, *star* patches show greater variability in compression, highlighting the role of structural geometry in mechanical performance. Hence, the structure of the HC regions of the patches influences the regime, the maximum degree, and the consistency of the patches' compression.

Directionality within the patch pattern is a critical determinant of mechanical response. The *hor line* and *ver line* patch types possess horizontal or vertical HC lines when viewed from the top (compare Figure 5a), and thus, their lines are in flow or perpendicular to the compressive flow, respectively. These *line* patches exhibit distinctly different compression behaviors despite having the same pattern rotated by 90°. *Hor line* patches with HC lines in flow demonstrate consistent and substantial reverse‐compression, while *ver line* patches with perpendicular lines display more variability and divergent compression regimes for individual patches. This suggests that directional alignment influences force distribution, making it an important consideration in material design.

Porosity only slightly affects the patches' compression behavior, though it does not alter the compression regime. Patches with different porosity levels compress similarly according to a linear regime. This uniformity is attributed to the consistent underlying patch pattern, indicating that porosity affects the flexibility and the local deformation capacity but not the fundamental compression response. Compared to the other patch types, the *porosity* patches were compressed at a significantly smaller pressure range since they started to intrude the 5 µm large pores of the filter beyond 40 mbar and thereby rupture at their LC regions. This squeezing and rupture behavior is indicative of a distinctly different mechanical behavior when approaching a constriction. However, their compression extent compares well with the other patches.

The direction of the applied force matters in determining the local mechanical characteristic of a patch, which highly depends on the patch geometry. A new chip design allowed for so‐called squeezing experiments. These allow to investigate the local compression of the patches in the y‐direction in addition to the longitudinal and overall compression in the x‐direction investigated via compression experiments. These mechanical characteristics are relevant for potential future applications, such as insertion of patches into a confined space. During squeezing experiments, the patches were squeezed through a 225 µm wide constriction of a microfluidic channel having the same width (y‐direction) and height (z‐direction) dimensions as the patches. Due to the height restriction, the patches could not buckle but remained in their 2D geometry. Figure 5c displays the squeezing of a *circle* patch. The width of the constriction corresponds to a quarter of the channel and patch width, resulting in a compression of the patches inside the constriction by 75%. The patches need to compress locally to surpass the constriction since the dimensions of the microfluidic channel hinder any third‐dimensional buckling or folding. To quantify the squeezing potential of the different types of patches, the pressure at which the patches break through the constriction was detected, as displayed in Figure 5e. Furthermore, the half‐through state is also depicted, which can be seen in Figure 5c at 1690 mbar for the shown *circle* patch. This state means the point where half of the surface area of the patch passes the constriction. Micrographs of the intrusion, the half‐through, and the breakthrough state of each patch type are displayed in Figures S13– S15 (Supporting Information), and a detailed discussion on the patches' squeezing behavior can be found in Section S6 (Supporting Information). Depicting the breakthrough pressures of the patches while squeezing through a constriction allows to compare the patches' local compression ability depending on their geometrical pattern.

The results show significant differences in breakthrough pressure between the patches of the *structure* group that have the same proportion of HC regions but different structural designs. The *star* patch type, with its thinner and more distinct HC regions, requires significantly less pressure to pass through the constriction compared to the *circle* patch type. This suggests that the structure of the HC regions, rather than just their proportion, is crucial for determining a patch's flexibility and ability to squeeze through tight spaces.

The *directionality* patch group shows how pattern directionality impacts squeezing behavior. The *ver line* patch type breaks through the constriction at a lower pressure than the *hor line* and the *reference* patches. Despite having identical patterns only differing in their directionality, the *ver line* and *hor line* patches differ in their breakthrough pressures, underscoring the significance of directionality within the patch pattern. The *reference* patch, composed entirely of HC regions, shows the highest resistance to breakthrough, often rupturing or getting stuck, proposing the proportion of the HC regions to determine the squeezing ability. Notably, the *star* patch type of the *structure* group, with a higher HC proportion (49%) than the *line* patches (29%), still required less pressure to pass through the constriction. Hence, in addition to the directionality and the HC region proportion, the structure and arrangement of HC regions are key factors in determining a patch's mechanical response.

The degree of third‐level porosity only slightly influences the patches' squeezing, but the pattern of the *porosity* group patches significantly impacts the squeezing compared to the other patches. In the *porosity* patch group, patches break through the constriction at much lower pressures (5–9 mbar) compared to the *structure* and *directionality* groups, which required at least 1000 mbar. This difference is due to the small size and low portion (7% or less) of HC regions of the *porosity* patches, making these patches highly flexible with large, interconnected LC regions. This becomes very apparent considering the patch *w/o pore* without pores. At this low portion of HC regions, the LC regions dominate the easy breakthrough, even if no third‐level porosity exists. Among the *porosity* patches, 44 µm *pore* patch type has a lower HC proportion (4%) than the two others and breaks through at a lower pressure, indicating that increased LC regions and greater distance between HC regions enhance flexibility. Interestingly, the presence of through‐pores in the 95 µm *pore* patch does not significantly affect its squeezing behavior, suggesting that the overall framework and HC region arrangement are more important than through‐pores in determining flexibility and squeezing.

**Figure 6:**
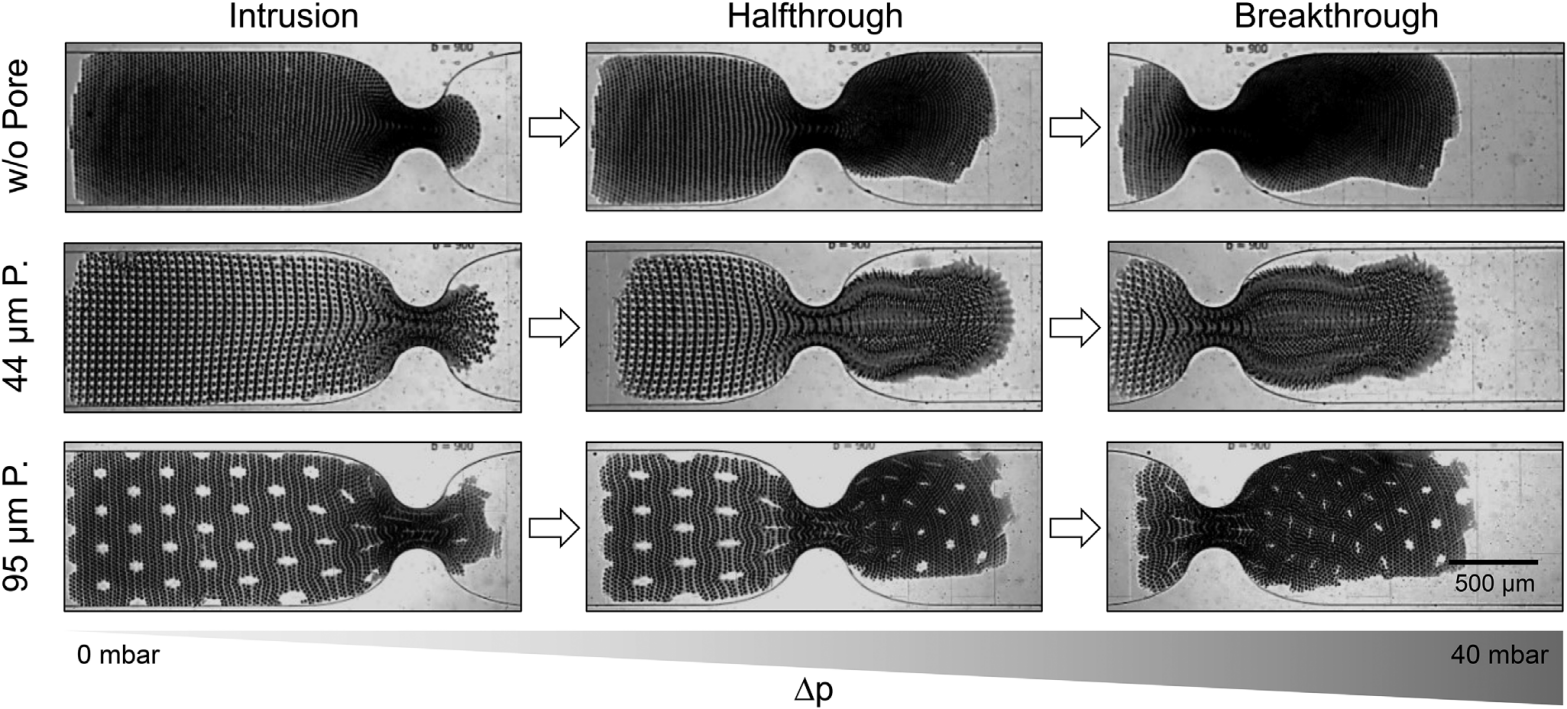
Brightfield images showing the squeezing states of the *porosity* patch types *w/o pore*, 44 µm *pore*, and 95 µm *pore*. Scale bar (500 µm) applies to all images. The shape of the through‐pores indicates local deformation states during the translocation through the constriction.

**Figure** **6** shows the translocation of the patches through the constriction and reveals particular features of the process of squeezing through. The through‐pores slowly deform toward the constriction, become highly elliptical up to disappearing visually in the constriction, and relaxing back into a close‐to circular shape when expanded into its new state. Whether or not the patches undergo plastic deformation in general or locally to different degrees cannot be identified from the current experiments.

In conclusion, the geometrical patterns of the patches determine their mechanical characteristics. As compression is favored at the LC regions within the patches, large and interconnected HC regions in a high proportion are expedient in increasing the mechanical stability of a patch. However, the proportion of HC regions shares its influence on the compression and squeezing ability with the HC structure and directionality. The induced third‐level porosity only slightly changes the compression of the patches, and the squeezing was also not significantly influenced by the through‐pores within the 95 µm *pore* patch. But, an induced third‐level porosity by changing the distance respectively arrangement of the HC regions leads to a slightly enhanced squeezing of the 44 µm *pore* patch. Hence, the proportion, structure, and directionality of the HC regions mainly influence the patches' mechanical characteristics regarding compression and squeezing, whereas the investigated third‐level porosity has less impact.

The high degree of freedom during hydrogel patch fabrication allows tuning of the mechanical requirements of the patches by adapting their geometrical patterns. The investigations of this section represent a foundation for predicting the mechanical characteristics of patches based on their geometrical pattern design. Shaping these patterns enables for varying mechanical requirements, such as adaptability to compression, mechanical anisotropy within patches, or varied flexibility. Using this novel fabrication procedure and combining the varying tuning factors presented in this work achieves a tailored, hierarchically structured hydrogel patch comprising cellular and gradient structural elements as desired for a targeted purpose. To engineer the mechanical properties of patches in the future based on bio‐inspired designs, simulation‐based hierarchical prototypes can serve as templates.^[^ ^37^, ^48^ ^]^

### 2.4 Cell Culture Scaffold with Tunable Morphology

Tissue engineering on scaffolds demands control over the mechanical properties and topography,^[^ ^5^, ^11^, ^12^, ^13^ ^]^ which can even, in some cases, determine cell fate.^[^ ^19^ ^]^ Owing to the patches' remarkable tunability in both form and mechanical attributes, they are well‐suited for application as cell scaffolds. To create a hydrogel scaffold suitable for cell culture, markers for cell adhesion have to be introduced to previously untreated hydrogels. Hydrogels created from synthetic polymers, such as PEGDA and PNIPAM, lack these binding sequences. In the case of PEGDA, glycidyl methacrylate (GMA) was used as a comonomer during fabrication and, thus, incorporated into the PEGDA‐based hydrogel patches to present epoxy groups throughout the hydrogel and on its surface. These epoxy groups are used to enable epoxy‐amine addition reactions with primary amines and, hence, to covalently bind to polypeptides such as fibronectin.^[^ ^15^, ^18^ ^]^ In contrast, the PNIPAM patches were only post‐functionalized with fibronectin using sulfosuccinimidyl 6‐(4'‐azido‐2'‐nitrophenylamino) hexanoate (sulfo‐SANPAH) as a bi‐functional crosslinker.^[^ ^49^ ^]^ With the functionalized patches, a concise cell study was performed as a proof‐of‐concept to investigate the reaction of cells to different topographies of the patches and their general suitability as structurally unique scaffolds.

**Figure 7:**
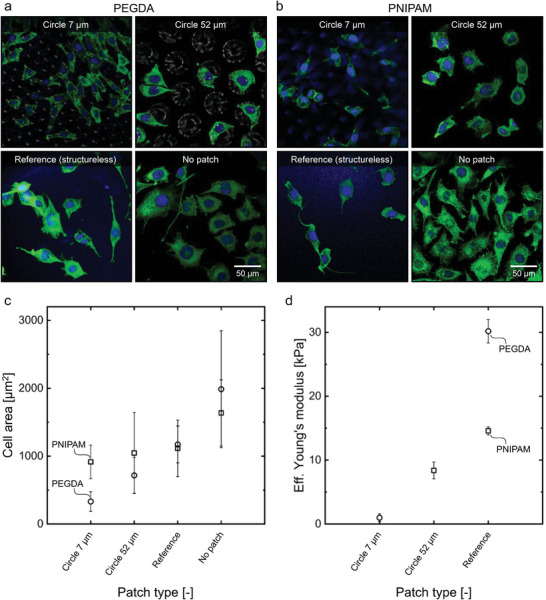
Cell cultivation (L929 mouse fibroblasts) on patches with varying structures and two different materials: a) PEGDA patches and b) PNIPAM patches with fibronectin coating. Patch patterns include circle‐shaped HC areas with diameters of 7 µm (*Circle 7* µm), with 52 µm diameters (*Circle 52* µm), and structureless (*Reference*) patches. Comparison of (a,b) visual cell appearance after two days of cultivation, c) cell area of isolated cells after this time, and d) Young's modulus of the patches. Scale bar (50 µm) applies to all micrographs.

**Figure** **7** illustrates the appearance of isolated L929 mouse fibroblast cells on specially designed patches made from PEGDA and PNIPAM materials, along with their corresponding cell areas and the patches' effective Young's moduli. The cell morphology and area vary depending on the pattern of the patches. In both material systems, PEGDA with GMA and NIPAM with BIS, the patch structure influences the cell area, as shown in Figure 7c. This effect was analyzed across different patch patterns, including hexagonally arranged circular HC regions with diameters of 7 µm (*circle 7* µm) and 52 µm (*circle 52* µm), structureless patches (*reference*), and a control without a patch (*no patch*).

Cell areas were calculated from microscopy images, depicted in Figure 7a for PEGDA patches and Figure 7b for PNIPAM patches. Cultivation was halted after two days to assess the morphology and area of isolated cells. Prolonged cultivation led to full overgrowth of the patches, causing cells to merge and lose their distinct morphologies.

In these images, after two days of cultivation, differences in cell areas and morphologies are evident. On the *circle 7* µm patch, featuring the smallest HC regions, cells appeared more elongated and smaller in both material systems compared to other samples with different structures. This morphological difference results in the smallest cell area for this sample (compare Figure 7c). Conversely, cells grown directly on the treated well bottom (*no patch*) exhibited the largest cell areas. Variations in cell area between patches likely correspond to the patch pattern, with smaller structures leading to smaller cell areas. This correlation is more pronounced for PEGDA patches, displaying a clear trend. Structured patches guide cell morphology and area, while structureless patches offer a flat surface for cell spreading.

The effects of patch pattern structure on cell properties are intricately linked with differences in material functionalization and variations in Young's modulus, making it challenging to fully disentangle their individual contributions. Notably, when comparing cell areas under control conditions (compare Figure 7c *no patch*), the subtle differences indicate the impact of functionalization. Specifically, the *no patch* condition shows a slightly larger cell area for the functionalization used in PEGDA patches compared to the sulfo‐SANPAH functionalization employed in PNIPAM patches. However, this difference is within the margin of error.

As depicted in Figure 7d, the effective Young's modulus appears to increase in tandem with cell area as the size of the HC regions within the patch structures increases, spanning a range of 1–30 kPa, which is suitable for various cell types.^[^ ^50^ ^]^ In addition to topographical cues, cells exhibit high sensitivity to the stiffness of scaffold materials, a factor that may confound the effects of structure on cell area.^[^ ^16^ ^]^ However, the findings from the *reference* patches suggest that Young's modulus alone cannot fully account for the observed differences in the cell area. Despite significant differences in Young's modulus between the structureless PEGDA and PNIPAM *reference* patches, the resulting cell areas are almost identical (compare Figure 7c *reference*). This indicates that while the stiffness of the patches may influence cell growth, topographical features appear to play a more pivotal role in determining cell area.

These observations underscore the need for further investigations to isolate the effects of patch topography from other influencing factors, providing a deeper understanding of the interplay between scaffold design and cell behavior. In conclusion, while functionalization and Young's modulus contribute to cell‐related parameters, the pattern of the patch structure emerges as the dominant factor within the scope of this study.

**Figure 8:**
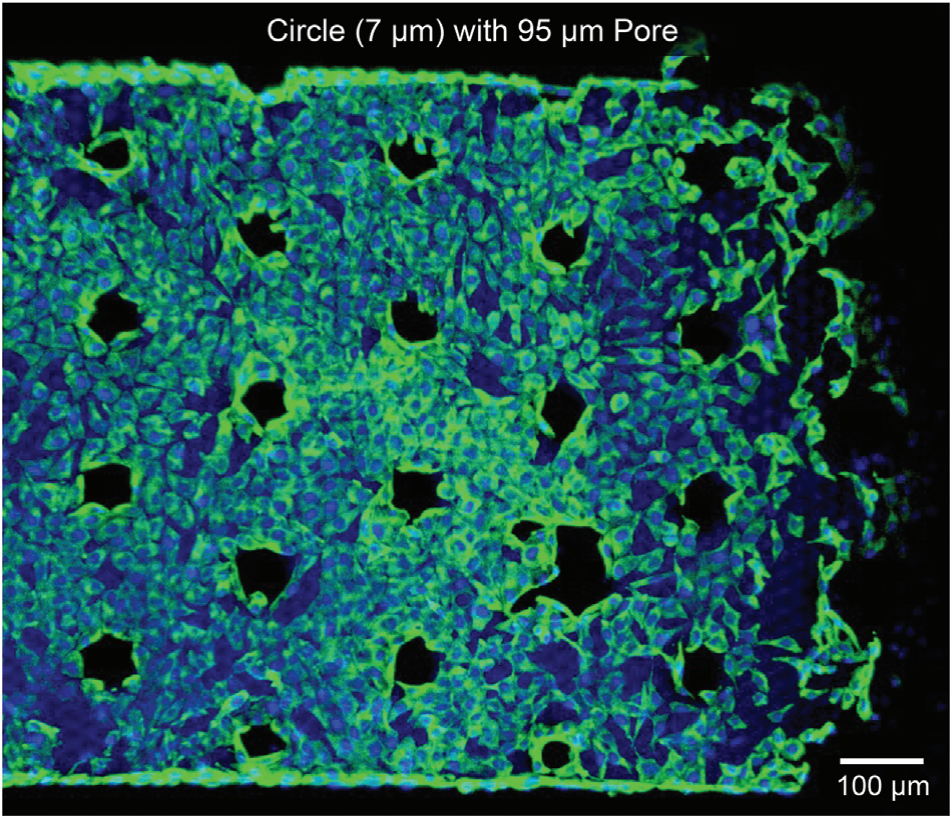
Cell cultivation (L929 mouse fibroblasts) on a *circle 95* µm *pore* patch with HC regions of 7 µm diameter in a hexagonal arrangement, regularly interrupted by through‐pores with 95 µm diameter after four days. The patch material is based on the PEGDA‐material system with the addition of GMA to enable fibronectin coating.

After four days of cultivation, cells proliferate extensively, covering nearly the entire patch surface and aligning according to the underlying patch pattern. As depicted in **Figure** **8**, cell growth is illustrated on a *circle 95* µm *pore* patch (compare Figure 4 95 µm *pore*). This patch features hexagonally arranged circular HC regions with a diameter of 7 µm and an edge‐to‐edge distance of 40 µm. The patch also incorporates through‐pores with diameters of 95 µm, arranged in a hexagonal pattern with inter‐pore distances of 164 µm.

Cells exhibit a highly organized arrangement around the through‐pores, effectively covering the entire patch surface area between them. These through‐pores may serve as channels for cell migration in stacked configurations of multiple patches, potentially enabling 3D tissue assembly with precisely tunable topography in future tissue engineering applications. Consequently, the hierarchical structuring of these patches offers a promising strategy for guiding and constraining cell propagation, facilitating the cultivation of tissue with predefined architecture and shape.

## 3 Conclusion

This work introduces the design, characterization, and potential applications of hierarchically structured and tunable hydrogels, called patches. These patches are hierarchical cellular materials fabricated in high throughput using projection lithography in stop‐flow mode. The patches are 80 µm in height and 2.25 × 0.9 mm in size, with intricate micrometer‐sized features. These features consist of highly crosslinked (HC) regions, which are patterned by the transparency mask during fabrication and, as a result, are adjustable in size, shape, and crosslinking degree, and are connected by low crosslinked (LC) regions. The HC/ LC geometry, the material properties, the porosity, the mechanical properties, and the functionalization of the patches can be customized.

This study on hydrogel patches identifies key parameters that influence patch formation, including radiant exposure, radiant power, and exposure time during polymerization for two material systems based on PEGDA and PNIPAM. An optimal diameter‐to‐distance ratio of HC regions of 0.4 or slightly greater is found for reliable patch formation, emphasizing the importance of geometrical patterns. By altering the mask design, the porosity and mechanical characteristics of the patches can be further fine‐tuned, enabling the formation of extra regions with through‐pores embedded into the patch, featuring accurately crafted topologies of spatially varied crosslinking densities. The proportion and connectivity of the HC regions determine the patches' mechanical characteristics, along with their structure and directionality. These investigations serve as a benchmark to design patches with desired characteristics.

The potential application of the patches as cell scaffolds is explored, demonstrating their suitability for tissue engineering and cell behavior studies. The customizable nature of the patches makes them ideal for observing cellular responses in different environments. Current research shows the sensibility of cells regarding stiffness and topology,^[^ ^13^, ^38^, ^51^ ^]^ which is especially of interest in larger scaffold defects with mesenchymal stem cells (MSCs),^[^ ^52^ ^]^ for mimicking complex organ architectures like gastrointestinal structures,^[^ ^53^, ^54^ ^]^ or for providing essential guidance in cartilage defects.^[^ ^55^ ^]^ By using the presented hydrogel patches, varying stiffnesses and a complex topology are enabled within a single hydrogel, which could gain traction in the presented research fields in tissue engineering. Beyond cellular applications, these patches have potential uses in non‐cellular fields, such as separation processes,^[^ ^56^ ^]^ soft robotics,^[^ ^41^ ^]^ and plant protection.^[^ ^57^ ^]^

## 4 Experimental Section

### Microfluidic Chip Fabrication

The microfluidic chips for the patch fabrication, the compression experiments, and the squeezing experiments were all produced via the same method. Therefore, masters for microfluidic chips were CAD designed (Inventor Professional 2023, Autodesk) and produced with a two‐photon lithography printer (Photonic Professional (GT) Printer, Nanoscribe GmbH) via dip‐in laser lithography. The remaining resin (IP‐S, Nanoscribe) was removed via two washing baths of 1‐methoxy‐2‐propyl acetate (10 min, ⩾ 99.5%, Sigma–Aldrich) and isopropanol (3 min, ⩾ 99.9%, Sigma–Aldrich), respectively. To harden the printed structure, the masters were cured under UV light (365 nm, 6 h). The microfluidic chips arise from subsequently casting the masters with poly(dimethylsiloxane) (PDMS) (Dow Corning, Sylgard 184 plus curing agent, 10:1 (w/w)). After curing (60 °C, overnight), the PDMS was detached and punctured at in‐ and outlet to achieve tubing holes. The PDMS was washed by sonication in isopropanol (5 min) and dried (RT, overnight). For the patch fabrication chips, a glass slide (52 × 76 × 1 mm, VWR) was coated with PDMS (200 µm thick layer) via a casting knife and cured (60 °C, overnight). Then, the PDMS form was bonded on the coated glass slide via oxygen plasma activation (TePla 100 Plasma System, PVA). An uncoated glass slide was used for the compression and squeezing chips.

### Reaction Solution Preparation

Two material systems were used to fabricate hydrogel patches. The first one is based on the pre‐polymer poly(ethylene glycol) diacrylate (PEGDA) (average molecular weight of M_n_ = 575 Da, with 400–600 ppm MEHQ as inhibitor, Sigma–Aldrich). The other one contains the monomer *N*‐isopropyl acrylamide (NIPAM) (98%, Acros Organics, recrystallized in hexane (99%, VWR)) and the crosslinker *N*, *N*'‐methylene bisacrylamide (BIS) (99%, Sigma–Aldrich). Both material systems were initiated via UV‐light (365 nm) and the photo‐initiator lithium phenyl‐2,4,6‐trimethyl‐benzoyl phosphinate (LAP) (⩾ 95%, Sigma–Aldrich). Water (HiPerSolv CHROMANORM, VWR) was used as a solvent solution. PEGDA patches that were used for cell cultivation, hereafter referred to as PEGDA‐GMA patches, were fabricated with the addition of the comonomer glycidyl methacrylate (GMA) (⩾ 97%, Sigma–Aldrich). For the preparation of the reaction solutions, depending on the material system, the monomer NIPAM or the pre‐polymer PEGDA (20 wt% each) were weighed in a vial (1.5 mL, Eppendorf) and water (77 wt% for PNIPAM, 79 wt% for PEGDA, or 78.8 wt% for PEGDA‐GMA, respectively) was added. The initiator LAP (1 wt%) was weighed in a separate light‐protected brown vial (1.5 mL, Eppendorf). In the case of the NIPAM material system, the crosslinker BIS (2 wt%) was weighed as well and added to the initiator vial. For the PEGDA‐GMA patches, the comonomer GMA (0.2 wt%) was treated equivalently. Afterward, the dissolved monomer or pre‐polymer solution was added to the initiator‐crosslinker, initiator, or initiator‐comonomer vial, respectively. This mixture was homogenized using a vortex mixer (Vortex Genie 2 Digital, Scientific Industries) (3000 rpm, 20 s). Subsequently, the vial was centrifuged (mini star silverline, VWR) (6000 rpm, 3 min) as the last preparation step of the reaction solution before hydrogel fabrication.

### Patch Fabrication

The hydrogel patches were fabricated via projection lithography inside microfluidic channels in a stop‐flow mode, known as stop‐flow lithography (SFL). A sketch of the setup is shown in Figure 1a. A transparency mask below the microfluidic channel shapes the UV light (365 nm, LZ1‐00UV00, LED Engin), which irradiates the reaction solution within the microfluidic channel (y: 900 µm wide, z: 80 µm high, x: 15 mm long). Thus, a photo‐induced radical polymerization starts, which results in the formation of hydrogels. All PNIPAM patches were fabricated with identical exposure parameters (200 mW, 750 ms, equals 38 mJ/mm^2^ ), except for the hydrogel formation study where the parameters were varied (compare Section 2.1). The PEGDA‐GMA patches for cell cultivation needed higher radiant exposure due to the comonomer addition (900 mW, 800 ms, equals 181 mJ/mm^2^ ). A continuous fabrication was achieved by applying an automated stop‐flow mode.^[^ ^45^, ^58^ ^]^ Thereby, the resting fluid was exposed to fabricate hydrogels. Afterward, a pressure difference (OB1, Elveflow) across the channel enabled the flow of the hydrogels out of the window of exposure by opening a solenoid valve (Type 6724, Bürkert). Next, the valve is closed again to stop the fluid, and the cycle is repeated. The setup is shown and detailed described in previous publications.^[^ ^45^, ^59^ ^]^ After fabrication, the reaction solution surrounding the hydrogels was exchanged against deionized water via dialysis (Zellutrans, MWCO: 12–14 kDa, 25 mm flat width, 20 µm wall thickness, Carl Roth GmbH) in a water bath (deionized, 10 L, 2 × 24 h). The purification of the patches used for cell cultivation was performed by sedimentation. Therefore, the fabricated patches within the unpolymerized reaction solution were light‐protectively sedimented for 5 min. Then, the supernatant reaction solution was discarded, and the sedimented patches were washed with fresh water. This sedimentation and washing procedure was repeated two times for a total of three cycles. Until analysis, all purified samples were stored at 4 °C.

### Polymeric Structure Analysis

To investigate the polymeric structure of the patches, the patches in water were transferred into a vial (DNA LoBind, 1.5 mL, Eppendorf), and excess water was carefully removed after sedimentation of the patches by using a pipette. The vial was sealed with sealing tape (parafilm, Heathrow Scientific) and perforated with a needle. The patches were frozen in liquid nitrogen by placing the vial into a filter cap bottle (150 mL, Christ) and attaching it to a freeze‐dryer (alpha 1‐4 LO plus, Christ) (2 h). The dried patches were imaged using a Schottky field emission electron microscope (FESEM) (SU5000, Hitachi).

### Compression and Squeezing Experiments

To investigate the mechanical characteristics of the patches, compression and squeezing experiments were performed for eight patch types (compare Figure 5a). Therefore, each patch in water was compressed against a filter structure or squeezed through a constriction inside microfluidic channels. First, each patch was flushed into the respective channel. Therefore, the inside of a tubing (FEP, 0.79 mm ID, 1.59 mm OD, Darwin) was wetted with an aqueous polyoxyethylene (20) sorbitan monolaurate (tween‐20) (Merck) solution (0.05 v%) to prevent the sticking of the patch. Via a connected syringe, the patch was aspirated into the tubing, and subsequently, the tubing was connected to the microfluidic chip and an inlet vial. The chip outlet was connected to an outlet vial. At the inlet as well as the outlet vial, pressure was applied via a pressure controller (OB1, Elveflow) to ensure a definite pressure difference across the microfluidic channel (compare Figures S5 and S12 for the setups, Supporting Information). Via minor pressure, the patch was flushed into the channel using the same aqueous tween‐20 solution (0.05 v%), and the orientation of the patch was checked to exclude previous compression or squeezing. Then, for both experimental kinds, a linear pressure ramp was applied on the patch (60 s), starting the compression or squeezing experiment. The pressure difference within the channel was set higher for the *structure* and the *directionality* patch types (0–1720 mbar) than for the *porosity* types (0–40 mbar). Both channels (y: 900 µm wide, z: 80 µm high, x: 8 mm long) had the height and width dimensions of the patches. The schematic structures of the microfluidic channels are depicted in Figure 5b,c. The filter structure at the end of the compression channel had 5 µm wide pores (100 µm distance to the next pore, equals 8 pores), which covered the whole channel height (z: 80 µm). These pores restrained the patch and let fluid stream through, allowing pressure to build‐up on the patch and compress it against the filter wall. The constriction within the squeezing channel had a width of 225 µm (y‐direction), corresponding to a quarter of the channel and patch width. The compression or squeezing was investigated via an inverse light microscope (DM IL LED, Leica). During each experiment, a video and the matching pressure values were recorded. Subsequently, a micrograph was extracted (every 1 s) and binarized. The surface area of the patch within each micrograph was determined by an algorithm (Python) that counts every pixel displaying the patch in the binarized image.

### Protein Functionalization of Hydrogel Patches

Hydrogel patches were transferred into 12 well cell culture slides (81201, Ibidi) and washed three times with sterile phosphate‐buffered saline (PBS) (1X, 200 μL, J62036.K3, Thermofisher) with resting times (5 min) between each washing step. For the functionalization of the PNIPAM patches, the PBS was aspirated and replaced by sulfosuccinimidyl 6‐(4'‐azido‐2'‐nitrophenylamino) hexanoate (sulfo‐SANPAH) (803332‐50MG, Sigma–Aldrich) dissolved in dimethyl sulfoxide (DMSO) (A3672,0100, ITW Reagents) (0.1 molL^−1^, 200 μL). The PNIPAM patches were exposed to UV light (365 nm, 5.2 mW/ cm^2^ , 14 min, Analytik Jena) before being washed three times with sterile PBS (1X, 200 μL) with resting times (5 min) between each washing step. For both patch kinds, PNIPAM and PEGDA‐GMA, the PBS was aspirated and replaced by a fibronectin solution (30 μg/mL, 200 μL, 10838039001, Sigma–Aldrich) before incubating the patches (37 °C, 24 h). After the incubation and before cells were seeded, gels were washed three times with sterile PBS (1X, 200 μL).

### Cell Cultivation

L929 mouse fibroblasts (85011425, Sigma–Aldrich) were cultivated in Gibco^TM^ high glucose Dulbecco's modified eagle medium (DMEM) (11965092, Thermo Fisher) supplemented with fetal bovine serum (FBS) (10 v%, 10‐FBS‐12F, Capricorn Scientific) and penicillin/streptomycin/amphotericin B (ABM) (1 v%, 15240062, Thermo Fisher). Cells were cultivated at 37 °C, 5% CO_2_, and 70% humidity. Cells were subcultured at 80% confluence, and the passage number was restricted to 25 subcultures. For a subculture, spent culture medium was aspirated, and cells were covered with trypsin‐like protease (TrypLE express enzyme, 12604013, Thermo Fisher) and incubated (37 °C, 5 min). Afterward, fresh culture medium (5–10 mL) was added, and cells were resuspended. The cell suspension was centrifuged (400 rcf^−1^, 5 min), and the supernatant was aspirated carefully after centrifugation. Cells were resuspended in fresh culture media and counted in a cell counting chamber with an automated cell counter (EVE, NanoEntek) in a mixture (50 v%) with the dye trypan blue (0.4%, NanoEntek). For experiments, a cell stock suspension (25000 viable cells /mL) was prepared, which was added (200 μL) to each sample.

### Immunofluorescence Imaging

After two days of cultivation, immunofluorescent staining was performed to subsequently determine the cell area of individual cells. For cell fixation, spent culture media was aspirated, and each well was washed once with PBS (1X, 200 μL). The PBS was aspirated, and paraformaldehyde (PFA) (4%, 200 μL, A3813,1000, ITW Reagents) was added and incubated (37 °C, 20 min). Each well was washed once with PBS (1X, 200 μL) before 4‐(1,1,3,3‐tetramethylbutyl)‐phenyl‐polyethylene glycol (Triton X‐100) (0.1 v%, 150 μL, 15 min, Sigma–Aldrich) was added to permeabilize the cell membranes. The wells were washed two times with PBS (1X, 200 μL) before a bovine serum albumin (BSA) blocking solution (1 v% in PBS, 150 μL, 126593‐100GM, Sigma–Aldrich) was added (RT, 30 min). The blocking solution was removed, and wells were washed two times with PBS (1X, 200 μL). F‐actin filaments were stained (60 min) with phalloidin‐iFluor 488 reagent (0.1 v%, 150 μL, ab176753, Abcam). Afterward, wells were washed with PBS (1X, 150 μL), before staining (5 min) with 4',6‐diamidino‐2‐phenylindole (DAPI) (0.4 v%, 150 μL, ab228549, Abcam). Wells were washed two times with PBS (1X, 200 μL) and stored at 4 °C under the exclusion of light until imaging. Imaging of the stained cell conditions was performed with confocal laser scanning microscopy (SP8 X, Leica) (excitation wavelength: 405 nm for DAPI, 488 nm for phalloidin).

### Cell Area Calculation

The cell area was determined with image processing software (1.54j, ImageJ). The scale bar in the exported microscopy images was used for software calibration. Images were transformed into 8‐bit versions, and one color threshold was applied for all images. The wand tracing tool was used to outline individual cells and measure them subsequently. 70 ± 28 cells of each condition were considered for the cell area mean values of each condition with a minimum of 27 cells per condition.

### Nanoindentation

Mechanical properties of hydrogels were determined using a nanoindenter (Pavone, Optics11Life). A cantilever probe with a spherical tip (49.5 µm radius, 0.45 N/m stiffness) was installed. Hydrogels were functionalized with fibronectin (30 μg/mL) like for cell cultivation and submerged in fresh culture media (1 v% ABM, 10 v% FBS in DMEM, 24 h). The nanoindenter probe was calibrated in culture medium, and all indentations were performed at the same conditions (RT, 1 μm/s speed, 7 µm indentation depth). Each hydrogel condition was measured at least three times, always at different locations. The effective Young's modulus *E*_eff_ was derived from the recorded indentation curves using the Hertzian contact model via the device‐associated data viewer software (V2.5, Optics11Life).

## Conflict of Interest

The authors declare no conflict of interest.

## Author Contributions

Lea Steinbeck contributed to conceptualization, data curation, formal analysis, investigation, methodology, project administration, supervision, validation, visualization, as well as writing the original draft and reviewing & editing the manuscript. Richard Paul was involved in conceptualization, data curation, formal analysis, investigation, methodology, project administration, supervision, validation, and writing – review & editing. Julia Litke, Isabel Karkoszka, and G. Philip Wiese contributed to investigation and methodology. John Linkhorst was responsible for conceptualization, project administration, and supervision. Laura De Laporte contributed to conceptualization, funding acquisition, and supervision. Matthias Wessling was involved in conceptualization, funding acquisition, project administration, resources, supervision, and writing – review & editing.

## Supporting information

Supporting Information

## Data Availability

The data that support the findings of this study are available from the corresponding author upon reasonable request.
