# Supplementary material for: Hierarchically Structured and Tunable Hydrogel Patches: Design, Characterization, and Application
Source: Small. 2024 Nov 20;21(3):2407311. doi: 10.1002/smll.202407311 (PMC11753498; doi:10.1002/smll.202407311)
Supplement: Supplementary file 1 — Supporting Information [file SMLL-21-2407311-s001.pdf]

NANO · MICRO
small

Supporting Information

for *Small*, DOI 10.1002/smll.202407311

Hierarchically Structured and Tunable Hydrogel Patches: Design, Characterization, and Application

*Lea Steinbeck, Richard Paul, Julia Litke, Isabel Karkoszka, G. Philip Wiese, John Linkhorst, Laura De Laporte and Matthias Wessling**

Hierarchically Structured and Tunable Hydrogel Patches: Design, Characterization, and Application

*Lea Steinbeck Richard Paul Julia Litke Isabel Karkoszka G. Philip Wiese John Linkhorst Laura De Laporte Matthias Wessling**

L. Steinbeck, R. Paul, J. Litke, I. Karkoszka, G. P. Wiese, J. Linkhorst[1], M. Wessling
Chemical Process Engineering AVT.CVT, RWTH Aachen University, Forckenbeckstraße 51, 52074 Aachen, Germany
Email Address: manuscripts.cvt@avt.rwth-aachen.de
L. De Laporte
Institute of Technical and Macromolecular Chemistry, RWTH Aachen University, Worringerweg 1-2, 52074 Aachen, Germany
L. De Laporte
Center for Biohybrid Medical Systems (CBMS), Advanced Materials for Biomedicine (AMB), Institute of Applied Medical Engineering (AME), University Hospital RWTH Aachen, Forckenbeckstraße 55, 52074 Aachen, Germany
R. Paul, L. De Laporte, M. Wessling
DWI – Leibniz-Institute for Interactive Materials e. V, Forckenbeckstraße 50, 52074 Aachen, Germany

S1 Patch Overview

Table S1 gives an overview of all patch types which are addressed in the main manuscript. Thereby, the dimensions of the used mask, as well as the dimensions within the patches, are listed. These dimensions vary by factor 5.46 as the optic-related characteristical value of the used SFL setup. The spot distances are indicated as center-to-center distances apart from specially labeled values that additionally indicate the edge-to-edge distances. All the mentioned patch types in Table S1 were fabricated out of poly(N-isopropyl acrylamide) (PNIPAM). Poly(ethylene glycol) diacrylate (PEGDA) patches were investigated with regard to their formation and to cell cultivation, comprising the following patch types: *circle (7 µm)/ w/o pore*, *circle (52 µm)*, *reference*, and *95 µm pore*.

The patches are classified according to their geometry and the associated investigations in the main manuscript. The *circle (7 µm)/ w/o pore* patch serves as the basic patch type and is considered in every investigation of the main manuscript. The patches of the *formation* groups were used to identify the ideal spot distance to diameter ratio in Section 2.1 (Figure 2, main manuscript) by adapting either the distance or the diameter of the circle-shaped spots. In Figure 2 (main manuscript), edge-to-edge distances are displayed. The group *structure* consists of four patch types introduced in Section 2.2 (main manuscript), shown in Figure 3 (main manuscript). They all have the same irradiated surface area, resulting in equal areas of highly crosslinked (HC) and low crosslinked (LC) regions. The 70 µm center-to-center distance and the hexagonal arrangement of the HC regions are equal as well, whereas the shapes of these regions differ. The patch types have slightly deviating diameters due to different surface-area-to-volume ratios of spot shapes. All characteristic variations between the *structure* patches can be directly related to their differing spot shapes, as all other parameters of the patches are identical. The *porosity* group compares the effect of the third level of porosity of patches, as discussed in Section 2.2 (main manuscript). The three studied patch types of this group were investigated in Figure 4 (main manuscript). The patches have an identical basic framework of HC regions, which are circle-shaped with diameters of 7 µm and are hexagonally arranged to each other with center-to-center distances of 26 µm. The *w/o pore* patch type has only two levels of porosity and serves as the reference of the group since it has no additionally induced pores. The other two patches have a third level of porosity each, which covers regions of 44 µm (*44 µm pore*) or 95 µm (*95 µm pore*) diameter, respectively. The last patch group concerns the induced *directionality*

[1]present address: Process Engineering of Electrochemical Systems, Technical University of Darmstadt, Otto-Berndt-Str. 2, 64287 Darmstadt, Germany

Table S1: Patch overview of all patch dimensions addressed in the main manuscript. Distances are indicated as center-to-center distances of the respective spots apart from specially labeled values that additionally indicate the edge-to-edge distances.

Group	Patch type	Spot shape	Spot arrangement	Spot diameter [µm] (mask/ patch)	Spot distance [µm] (mask/ patch)	Proportion of highly-cross-linked area [%]	Occurrence (section/ figure)
Formation, Porosity	Circle (7 µm)/ w/o Pore	circle	hexagonal	40 / 7	140 / 26 edge: 100/ 18	7	2.1, 2.2, 2.3, 2.4 / 1, 2, 4, 5, 6
Formation	Circle D90	circle	hexagonal	40 / 7	90 / 16 edge: 50/ 9	18	2.1 / 2
	Circle D240	circle	hexagonal	40 / 7	240 / 44 edge: 200/ 37	3	2.1 / 2
	Circle d30	circle	hexagonal	30 / 5	130 / 24 edge: 100/ 18	5	2.1 / 2
	Circle d60	circle	hexagonal	60 / 11	160 / 29 edge: 100/ 18	13	2.1 / 2
Structure	Circle (52 µm)	circle	hexagonal	282 / 52	382 / 70	49	2.2, 2.3, 2.4 / 3, 5, 6
	Square	square	hexagonal	250 / 46	382 / 70	49	2.2 / 3
	Snowflake	snowflake	hexagonal	270 / 50	382 / 70	49	2.2 / 3
	Star	star	hexagonal	380 / 70	382 / 70	49	2.2, 2.3 / 3, 5
Porosity	44 µm Pore	circle	hexagonal	40 / 7	140 / 26	4	2.2, 2.3 / 4, 5
	95 µm Pore	circle	hexagonal	40 / 7	140 / 26	7	2.2, 2.3, 2.4 / 4, 5, 7
Directionality	hor Line	rectangle	linear	40 / (2250 x) 7	140 / 26	29	2.3 / 5
	ver Line	rectangle	linear	40 / 7 (x 900)	140 / 26	29	2.3 / 5
	Reference	-	-	-	-	100	2.3, 2.4 / 5, 6

of patches. Therefore, two patches with 7 µm thick line-shaped HC regions with distances of 26 µm are compared. Viewed from the top view, the direction of the lines of the *hor line* patch is horizontal and thus along the longitudinal x-axis of the patch. The *ver line* patch has vertical lines that are orthogonal to its longitudinal axis and thus along its y-axis. As a *reference*, a patch type is used, which consists only of the HC region without any mask pattern applied during fabrication. Thus, this patch type has no directionality and serves as the reference for all fabricated patches since no pattern and thus no differentiation between crosslinking, porosity, or stiffness appears within this patch type. The influence of the respective specified differences of the *structure*, *porosity*, and *directionality* groups on their mechanical characteristics are compared in Section 2.3 (main manuscript).

S2 Hydrogel Formation

S2.1 Formation Events

The patch formation process is schematically displayed in **Figure S1**a. During projection lithography, the transparency mask patterns the light, which reaches the reaction solution and induces free-radical photopolymerization. Beyond that, partial polymerization occurs beyond the irradiated regions, mainly caused by diffusion and light scattering. Patches arise when a coherent polymer network forms beyond the distinct regions defined by the transparency mask due to these attenuated polymerizations. Whereas HC regions form at the directly irradiated regions, LC regions develop between them. Patch formation is

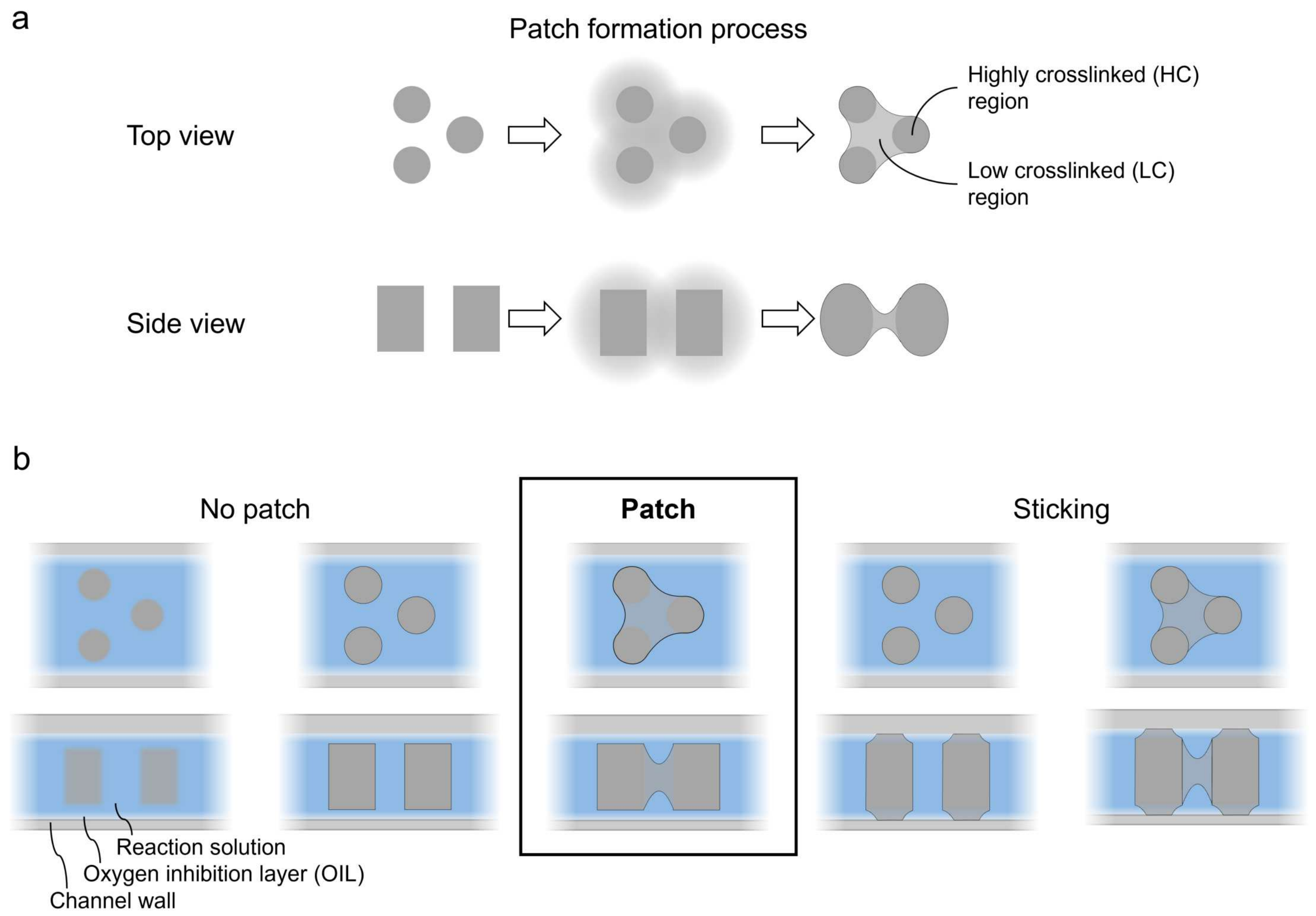

Figure S1: (a) Process of patch formation and (b) possible formation events during light-induced free-radical hydrogel polymerization.

only one possible formation event that can occur during projection lithography. The formation events can be divided into three main groups: *no patch*, *patch*, and *sticking*. For simplification, only one formation event per group is visually presented in the main manuscript (compare Section 2.1). Figure S1b shows both subevents of the groups *no patch* and *sticking*, each, that were only briefly addressed in the main manuscript. Compared to the *patch* formation, the polymer network forming during the *no patch* formation event only occurs at the directly irradiated areas. Either the networks are stable, resulting in uniformly crosslinked hydrogels in the shape of the irradiation spots of the mask (compare Figure S1b, *no patch*, right), or the induced radiant energy is insufficient to form stable polymer networks (compare Figure S1b, *no patch*, left). Thereby, uniformly crosslinked refers to only one induced degree of crosslinking within the hydrogels. However, there might be minor variations within the hydrogels due to the method, for example, because of providing the associated irradiation from one channel side and setting the present light

focus in the middle of the channel height. These minor deviations, however, are considerably marginal compared to the induced levels of crosslinking of the patches by the mask pattern (compare Figure 3, main manuscript). During the *sticking* event, uniformly crosslinked hydrogels can arise as well. However, all polymerized hydrogels of this event stick to the microfluidic flow channel. This sticking can occur for uniformly crosslinked hydrogels (compare Figure S1b, *sticking*, left) and for patches with HC and LC regions (compare Figure S1b, *sticking*, right). *Sticking* occurs when the oxygen inhibition layer (OIL) is depleted, commonly resulting from a high radiant power at the irradiated regions. The local radiant power can be increased by the light source or by greater irradiation spot diameters or decreased spot distances, resulting in *sticking* being more likely (compare Figure 2, Section 2.1, main manuscript). Since such irradiation spot variations additionally favor *patch* formation, the occurring *sticking* more likely is a *sticking* of a patch instead of a *sticking* of uniformly crosslinked hydrogels. Hence, which of the *sticking* subevents occur mainly depends on the mask pattern.

S2.2 Material System Differences

The reaction solutions of the PEGDA-based and the NIPAM-based material system investigated in Section S2.1, main manuscript, were examined regarding their rheology. **Figure S2** shows the storage G' and the loss modulus G" of both solutions over the time. After 60 s of the measurement, UV irradiation started. While the moduli at the end of the measurements are similar for both material systems, the characteristics differ directly after irradiation.

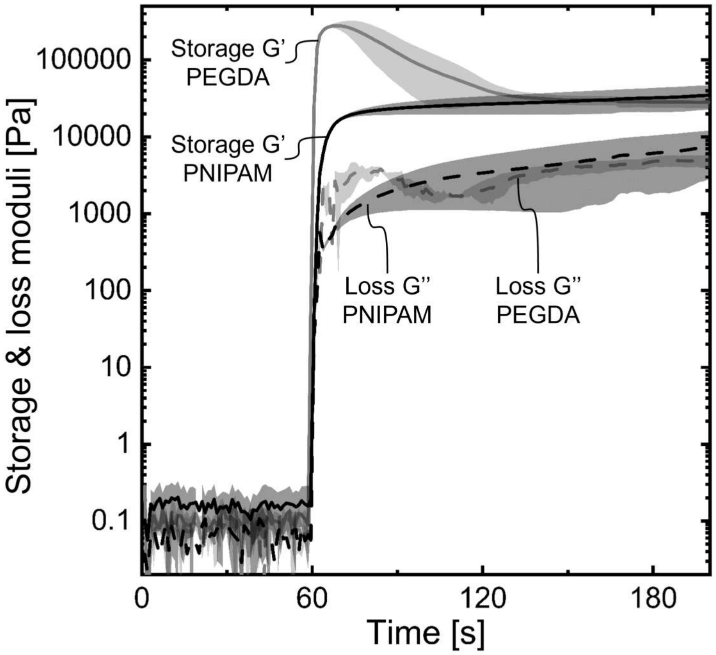

Figure S2: Storage modulus G' (solid line) and loss modulus G" (dashed line) of a PEGDA (gray) and a NIPAM-based (black) material system during UV-irradiation starting at 60 s.

Rheometry Experimentals: Reaction solutions used for patch fabrication were characterized using rheometry (Discovery HR-3, TA instruments). A cone plate geometry (20 mm diameter, 2.0°) was used. Experiments were performed in dark conditions at room temperature with a stage temperature of 25 °C. A UV LED accessory (533219.901, TA Instruments) was installed to initiate the polymerization during measurements. All rheological data was collected by placing the reaction solution (80 µL) on the UV stage (54 µm sample gap distance). Stress and viscosity were monitored along with increasing shear rate (0.1 to 100 s^{-1}). Storage and loss modulus were determined at a constant oscillation strain (1%) and frequency (1 Hz) process. 60 s after the beginning of the measurement, UV irradiation started (5 mW cm^{-2}) until the end of the measurement.

S3 Porous Structure Investigation

Figure S3 shows field emission scanning electron microscopy (FESEM) images at three different magnifications of a *circle 52 µm* and a *star* patch, using freeze-drying as sample preparation method.

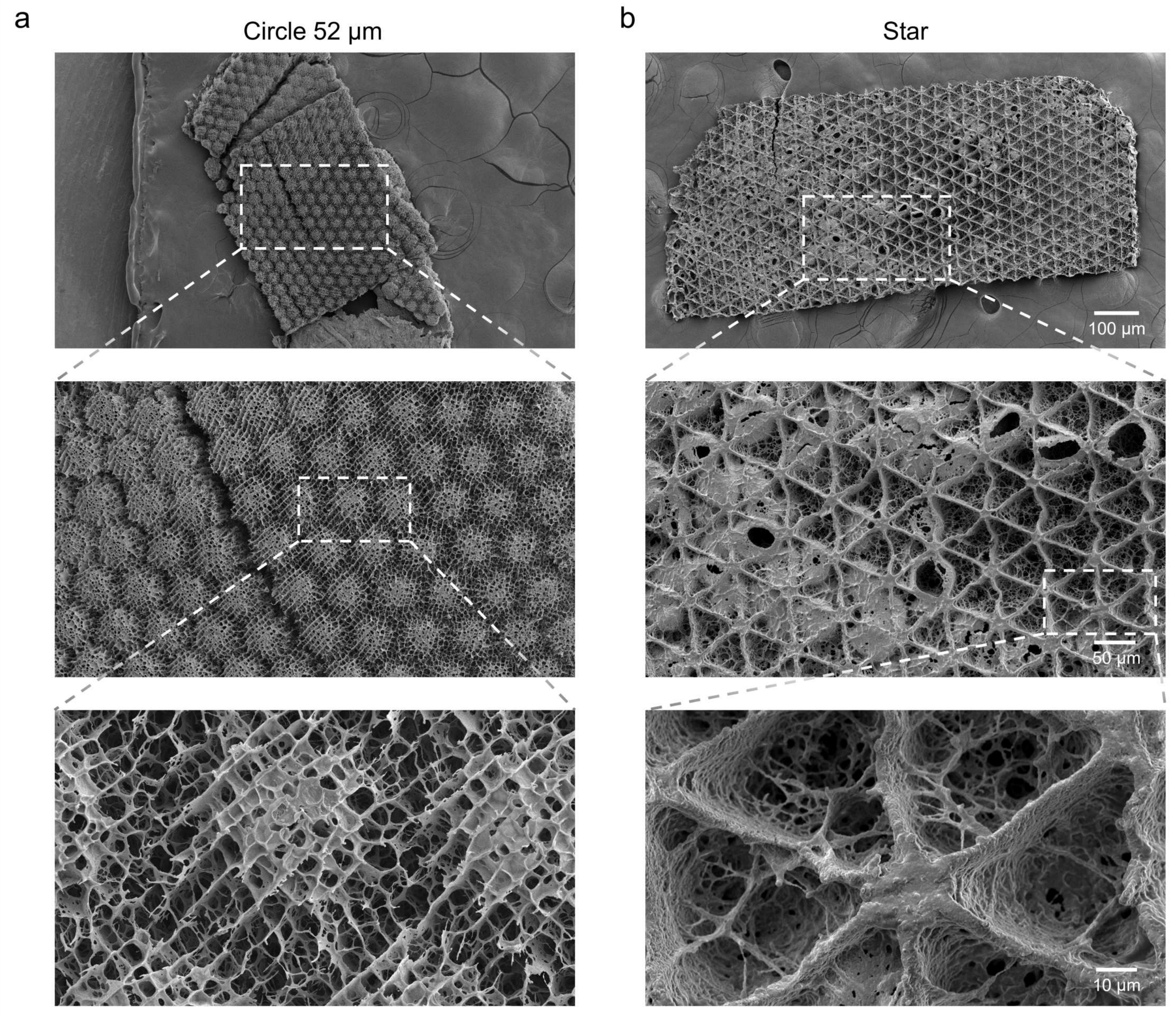

Figure S3: Field emission scanning electron microscopy (FESEM) images of patches with (a) circle-shaped (*circle 52 µm*) or (b) star-shaped (*star*) irradiation spots after freeze-drying.

S4 Pipette Aspiration

To investigate the mechanical characteristics of the patches, their folding capability was visualized by aspirating the patches with a pipette. These experiments highlight which regions within the patches are softer and able to be folded. However, this kind of experiment does not allow a well-founded scientific comparison of the patches since the equality of the experimental parameters cannot be ensured between single experiments. Hence, the experiments are hardly reproducible, and the folding of the patches represents only a tendency instead of definite investigations. Reproducible investigations of the mechanical characteristics of the patches were achieved by compression and squeezing experiments, shown in Figure 5

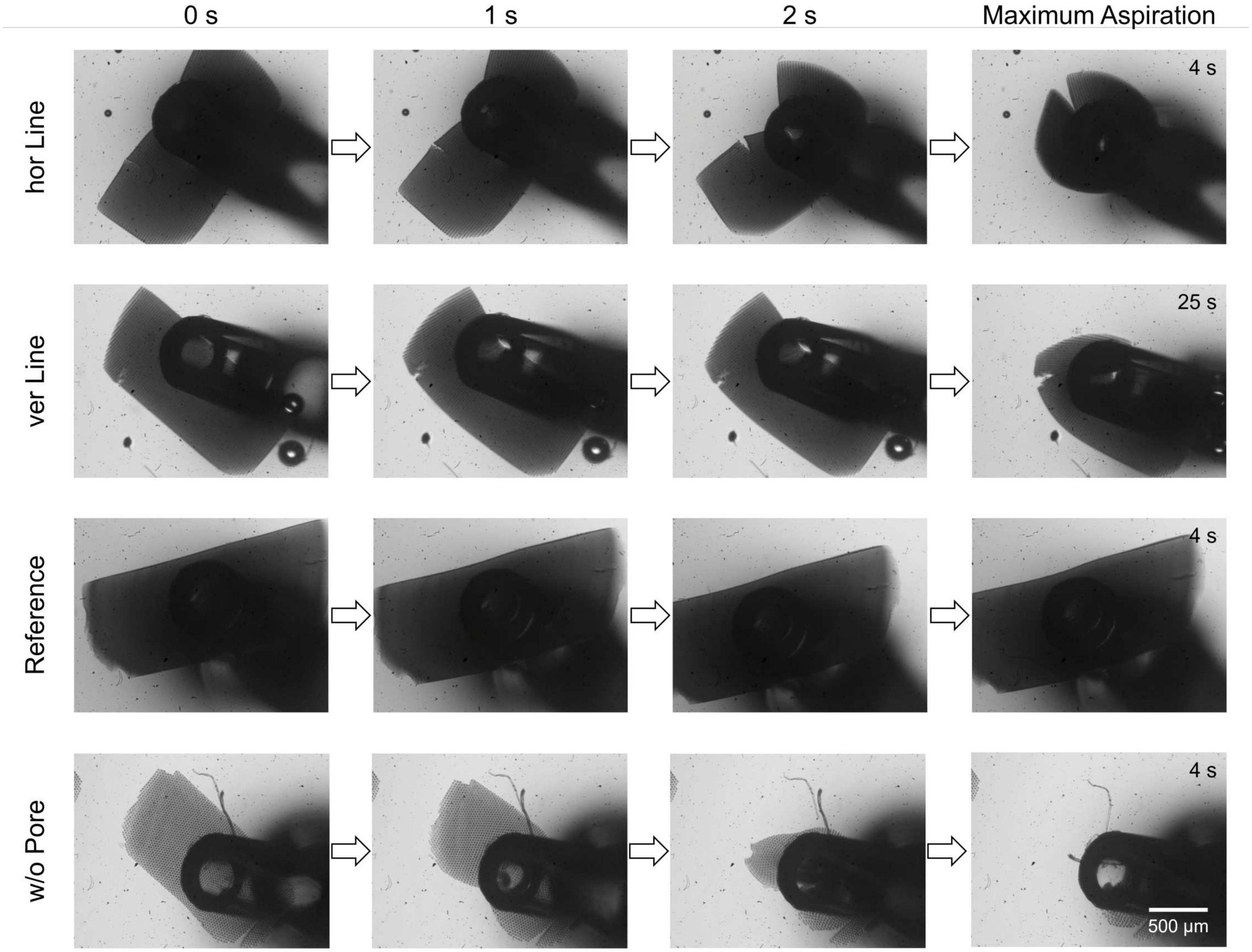

Figure S4: Pipette aspiration of all *directionality* group patches and the basic patch type *circle (7 µm)/ w/o pore*, showing their folding capability and flexibility. Scale bar (500 µm) applies to all images.

and discussed in Section 2.3 (main manuscript). In contrast to the aspiration experiments, the compression and squeezing experiments are reproducible, and the local folding behavior during squeezing is verifiable due to identical and measurable parameters of the experiments. However, the folding in the squeezing experiments is limited to the microfluidic flow channel that has the same dimensions as the uncompressed patch and a directed folding inside a constriction. Due to the circle-shaped pipette tip, the folding during aspiration is not directed. Hence, the aspiration inside a pipette tip provides additional information on the mechanical characteristics of the patches. The pipette aspiration of four of the patches used in the compression and folding experiments is shown in **Figure S4**. Here, all three patches of the *directionality* group, *hor line*, *ver line*, and *reference*, and the basic patch type *circle (7 µm)/ w/o pore* are pictured. The *hor line* and *ver line* patch showed a significantly different folding behavior during aspiration, despite their almost identical formation parameters. The only difference between these patches is the direction of their HC lines. This difference causes a considerably stronger and faster folding of the *hor line* patch within the pipette tip. Hence, the folding during pipette aspiration is highly pattern-dependent as well. The *reference* patch that comprises no pattern but one coherent HC region did not fold at all since the diameter of the pipette tip was smaller than the length and width of the patches. In contrast, the *w/o pore* patch completely enters the pipette tip. This patch is highly flexible due to its small circle-shaped HC regions and their hexagonal arrangement. Since the folding of the patches is favored along their less

stiff LC regions, a hexagonal arrangement results in great folding flexibility. In addition, a low proportion of HC regions facilitates the folding.

Pipette Aspiration Experimentals: For the pipette aspiration experiments, a single patch was transferred onto a microscope slide (26 x 76 x 1 mm, VWR) within a water droplet. The slide was placed under an inverse light microscope (DM IL LED, Leica), and a high-speed camera (Phantom VEO 640 L, Metek) was mounted to record the experiments. For aspiration of the patch, a pipette tip was wetted with a tween-20 solution (5 v%), placed over the sedimentated patch, and aspirated it.

S5 Compression of Patches

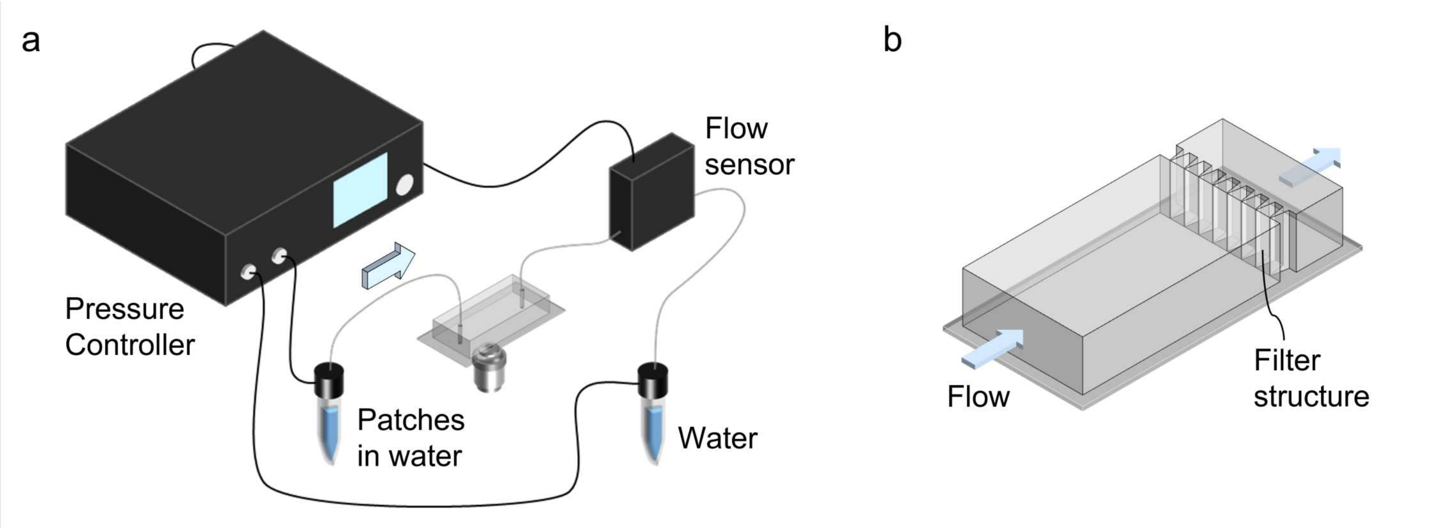

Figure S5: Schematic (a) setup to study the compression of patches and (b) detailed microfluidic channel scheme with displayed filter structure.

Figure S5 shows a scheme of the setup and the microfluidic channel which were used to investigate the compression of the patches. A filter structure within the channel retained the patches while the surrounding fluid passed. Thus, the patches were compressed against this filter.

S5.1 Filter Intrusion

Above a pressure difference of 40 mbar, the patches of the *porosity* group intrude into the filter structure of the microfluidic channel, whereas their LC regions rupture. The HC regions seemed to be stable against rupture by the applied pressure in contrast to the LC regions connecting them. Since the HC regions of these patch types are about 7 µm in diameter, they can be pressed through the filter in contrast to the larger HC regions of the *structure* patches or the *reference* patch. The line-shaped HC regions within the *hor line* and *ver line* patches have the same width as the *porosity* patch circle diameters of 7 µm (compare Table S1). However, the pattern of these patches provides greater stability by the length of these regions and the decreased flexibility of the *line* patch types, preventing the patches from intruding into the pores of the filter. Moreover, the lines within the *ver line* patches do not surpass the filter due to their orthogonal directionality towards the filter. In contrast, the HC regions of the *porosity* patch types are small, and their patterns feature one huge interconnected LC region, enclosing the circle-shaped HC regions. As a result, the *porosity* patch types were compressed with a maximum pressure difference of 40 mbar instead of 1720 mbar like all other patch types.

S5.2 Visual Compression Investigation

The **Figures S6, S7, and S8** show the compression of the patch types of the *structure* group and all patch types of the *directionality* and *porosity* group. Their uncompressed, maximal compressed, and end states are displayed each.

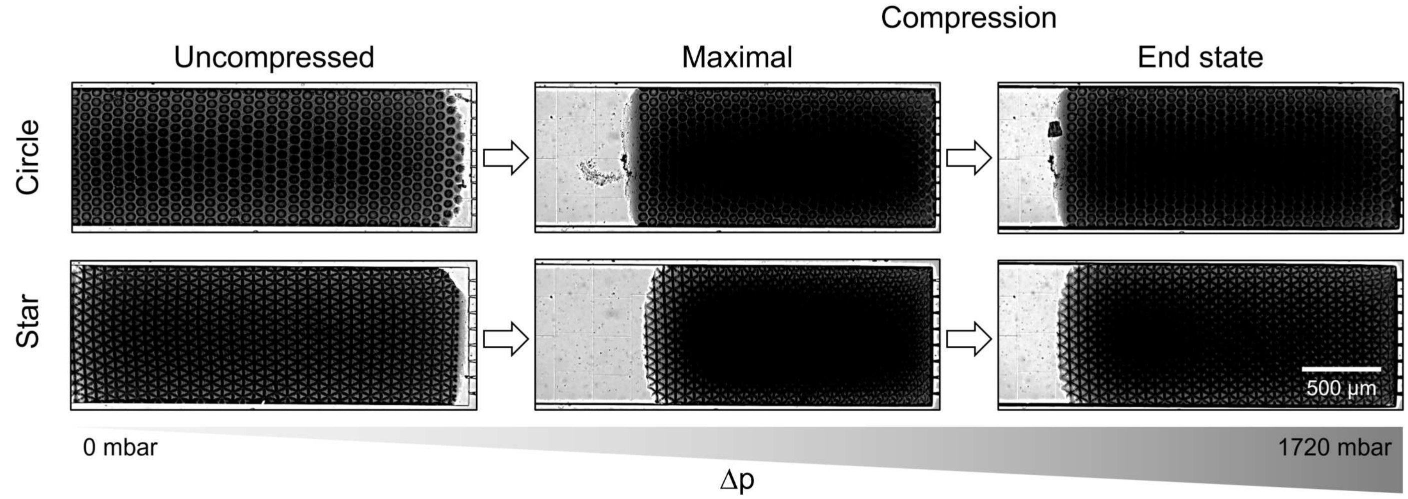

Figure S6: Compression states of the *structure* patch types *circle* and *star*. Scale bar (500 µm) applies to all images.

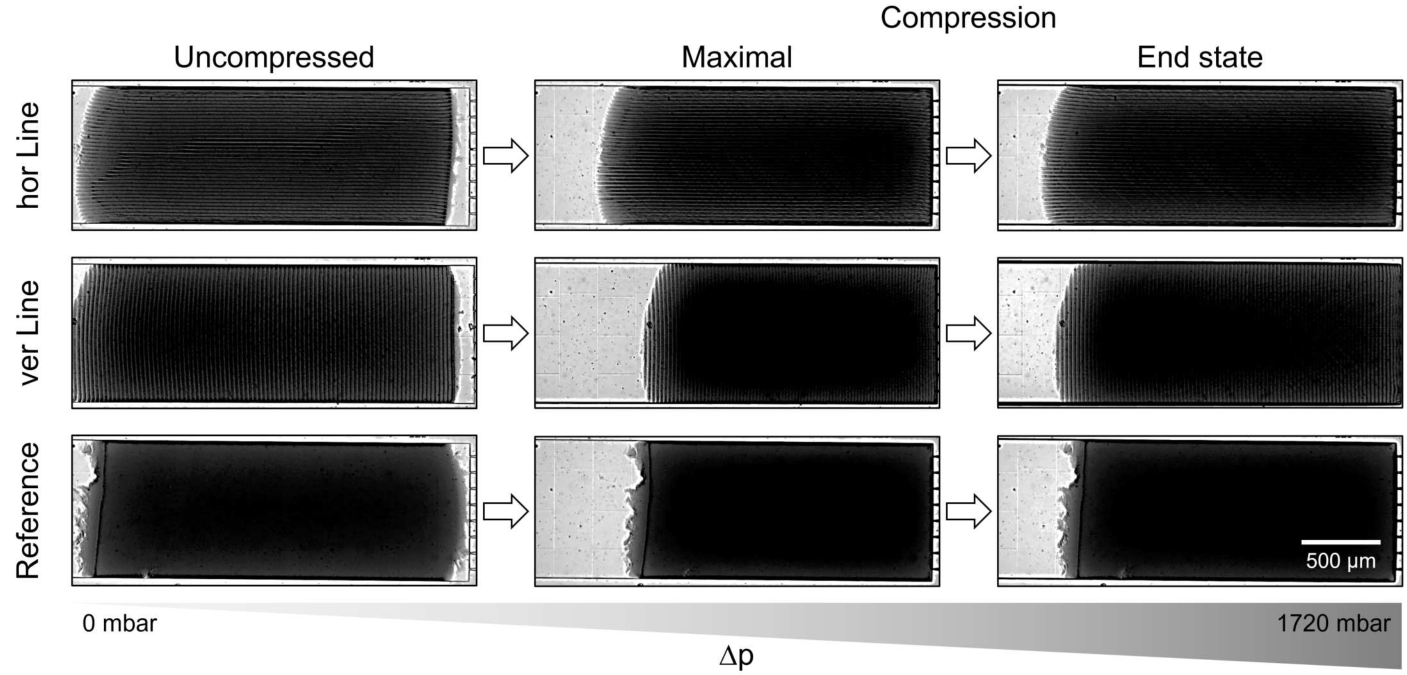

Figure S7: Compression states of the *directionality* patch types *hor line*, *ver line*, and *reference*. Scale bar (500 µm) applies to all images.

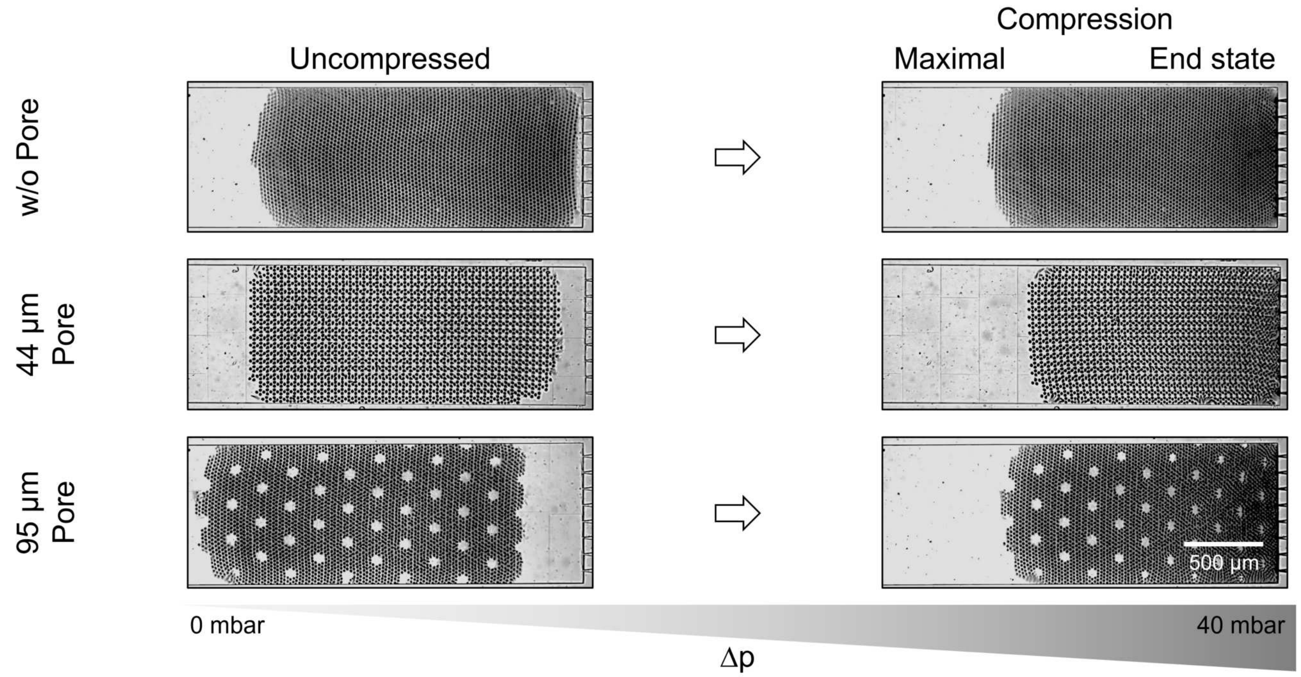

Figure S8: Compression states of the *porosity* patch types *w/o pore*, *44 µm pore*, and *95 µm pore*. The maximal and the end state compression are equal for these patch types. Scale bar (500 µm) applies to all images. Scale bar (500 µm) applies to all images.

S5.3 Compression Values

Table S2 shows the normalized area of the patches and the respectively applied pressure at the maximum compressed state and the end state. Furthermore, the reversed surface area is listed. These normalized areas and the pressure at maximum compression are additionally depicted in **Figure S9**.

Table S2: Compression values.

	Maximum compression		End state compression		Reversion
Patch type [-]	Applied pressure [mbar]	Normalized surface area [-]	Applied pressure [mbar]	Normalized surface area [-]	Reversed surface area [-]
Circle	932 ± 293	0.80 ± 0.04	1721 ± 0	0.84 ± 0.03	0.27 ± 0.08
Star	881 ± 285	0.74 ± 0.04	1719 ± 1	0.82 ± 0.04	0.30 ± 0.13
hor Line	675 ± 62	0.82 ± 0.05	1720 ± 0	0.93 ± 0.02	0.59 ± 0.12
ver Line	926 ± 471	0.79 ± 0.07	1720 ± 1	0.85 ± 0.05	0.39 ± 0.12
Reference	900 ± 428	0.79 ± 0.07	1720 ± 1	0.84 ± 0.08	0.48 ± 0.07
w/o Pore	39.6 ± 0.6	0.83 ± 0.06	39.6 ± 0.6	0.83 ± 0.06	-
44 µm Pore	39.7 ± 0.5	0.80 ± 0.05	39.7 ± 0.5	0.80 ± 0.05	-
95 µm Pore	40.5 ± 0.5	0.86 ± 0.03	40.5 ± 0.5	0.86 ± 0.03	-

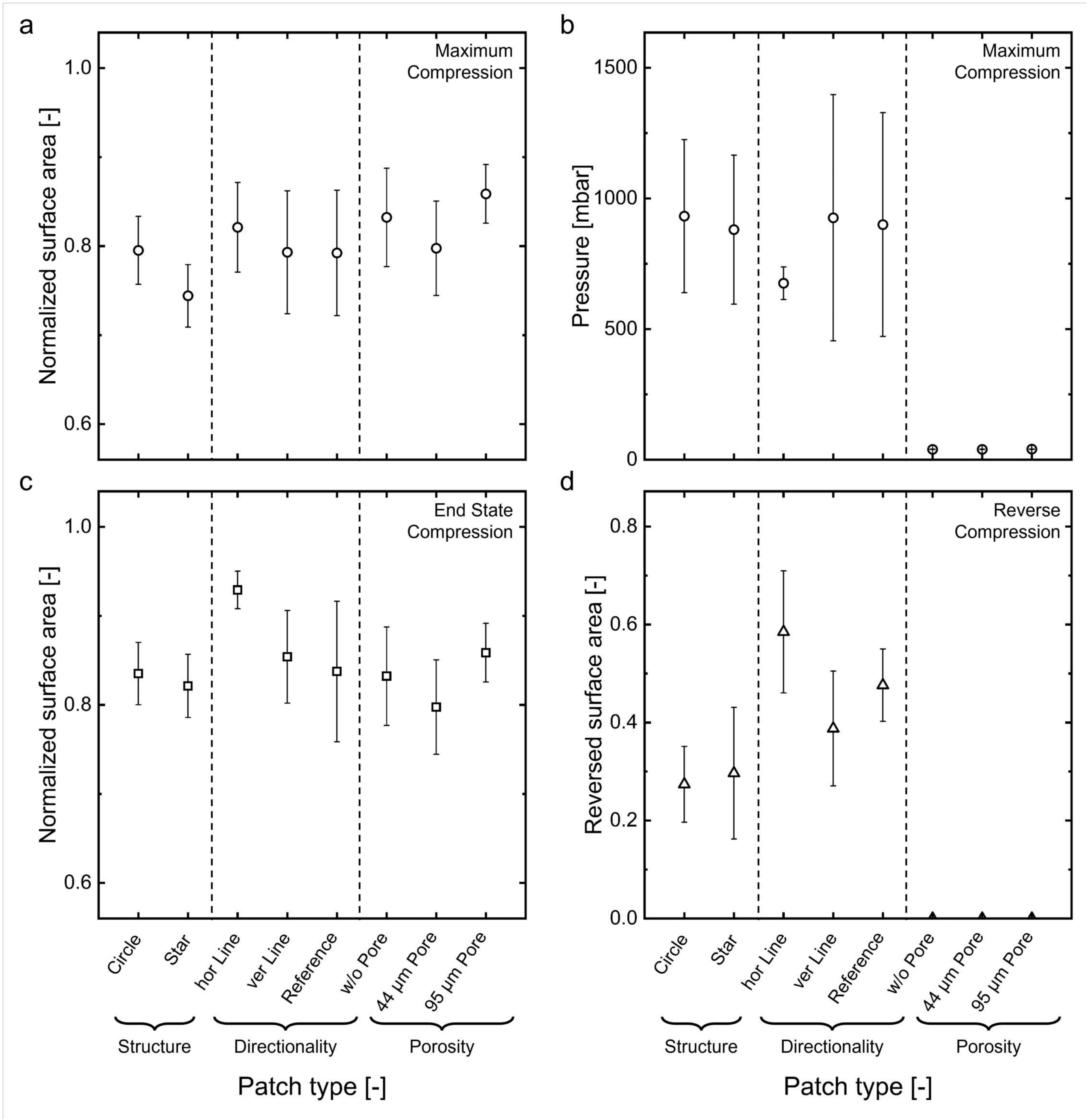

Figure S9: Compression values showing the normalized surface area of two *structure*, all *directionality*, and all *porosity* patch types for (a) the maximum compressed and (c) the end state. (b) The applied pressure at the maximum compressed state is displayed, as well as (d) the reversed surface area.

S5.4 Compression Regimes

When regarding the normalized surface area over the applied pressure during compression, as in Figure 5d in the (main manuscript), three different compression regimes occurred: linear, plateau, and reverse compression.

Linear Compression

According to the linear regime, the area of the patch decreases linearly over increasing pressure. The compression of all *porosity* patches follows this regime, as well as single patches of the *ver line* and *reference* patch types. This regime implies that the maximum compression of the patch is not reached within the applied pressure range as the compression continues instead of reaching a maximum value. For the *porosity* patch types, this implication is assured: Their actual maximum compression could not be detected since these patches begin to penetrate the filter structure when the pressure range of 40 mbar is exceeded. The micrographs of the compression in the end state shown in Figure S8 are in broad areas similar to the uncompressed state, confirming that the patches are not maximally compressed.

Plateau Compression

The plateau regime is very similar to the linear one. However, the patch area only linearly decreases until it reaches a constant minimum surface area and thus the true maximum compression of the respective patch in constrast to the first linear compression regime. Thereby, a plateau emerges in the compression regime instead of a linearly decreasing straight over the entire pressure range. No patch type shows this regime exclusively for all observed patches. Instead, this regime was present for single *circle*, *ver line*, and *reference* patches. The other patches of these kinds show either the linear or the reverse compression regime.

Reverse Compression

The reverse compression regime is characterized by an initial strong decrease in the patch area over the applied pressure followed by a reincrease. The reverse compression indicates how much of the maximum compressed area has been relaxed again. The reverse surface area is depicted as the surface area difference between the maximum compressed and end state divided by the difference between the uncompressed and maximum compressed state, according to **Equation S1**:

$$A_{Reversed} = \frac{A_{Endstate} - A_{Maximum}}{A_{Uncompressed} - A_{Maximum}} \tag{S1}$$

Reversed surface area calculations only referred to the patches which follow the reverse compression regime. Thus, not necessarily all patches of a patch type are considered in the calculations of the mean reversed surface area.

The reverse compression regime was observed for all patches of the *star* and the *hor line* patch types, as well as for some patches of the *reference*, *ver line*, and *circle* patch types. These reverse compressing patch types are from the groups *structure* and *directionality* and consist of a minimum amount of 29% out of HC regions in relation to the total patch surface area. In contrast, for the *porosity* patch types, the HC regions constitute only 7% of the patch area or less and these patch types do not follow the reverse compression regime. Thus, a high proportion of HC regions most likely result in the reversed compression behavior.

A likely assumption regarding the mechanism is that the reverse compression is caused by strongly compressed patches blocking the channel and the filter structure and, thus, shifting the forces applied within the channel. This blockage hinders the fluid from passing through the channel. The fluid flow, as the main force that acts on the patch, points in the x-direction towards the filter structure during the microfluidic compression experiments. However, the fluid most probably mainly flows around the patch and thus along the channel walls since the patch occupies the channel's mid. Hence, this surrounding flow might additionally apply a force on the patch from the channel walls towards the channel mid in y- and z-direction. With

stronger compression of the patch, the fluid space at the channel walls around the patch gets narrowed. As a result of the limited space, the force applied by the fluid flowing around the patch might be increased until this force is stronger than the force in the flow direction toward the filter structure. Consequently, the compression direction might change, and the patch is compressed towards the channel mid, resulting in an increasing normalized surface area in the xy-plane, as reported in the measurements. This potential shift of forces might be an explanation for the reversed compression of the patches according to the reverse compression regime. When looking at the micrographs taken during different states of compression, this assumption becomes even more likely (compare Figures S6 and S7). During maximum compression, the center of the patches in the top view (xy-plane) is significantly darkened, with only thin, less dark areas at the patches' edges. In the end state, however, the patches appear significantly lighter, particularly at the edges of the channel, which indicates less compression at these locations. This observation supports the assumption that the fluid flows past the side of the patches at the channel walls and that the blockage is decreased at these locations after the reversed compression. Patch patterns with large and continuously connected HC regions seem to increase the degree of reverse compression. Such patch patterns most likely increase the resistance of the flow through the channels' mid and favor the flow around the patch. In particular, the *ver line* and *reference* types represent flow obstacles as the connected HC regions cover the entire channel width and are orthogonal to the flow. According to the hypothesis of how the reverse compression might arise, the surrounding flow and the resulting force driving the reverse compression might be increased for the *directionality* patch types. In addition, the impact of this force might be increased due to the interconnection and extension of the HC regions, affecting the whole patch to a greater extent. Thereby, the HC regions of the *hor line* patches have more space to evade in the favored x-direction to especially recompress the HC regions since the channel length is only limited on one side by the filter. The favored evasion direction of the *ver line* patches is limited by the channel width in the y-direction. This might explain the favored reversed compression of the *hor line* patches in comparison to the *ver line* and *referene* patches, as well as the *directionality* group's strong reverse compression in general.

S5.5 Compression Behavior

Structure Patches Compared

Although the *circle* and the *star* patch types of the *structure* group share the same ratio of HC and LC regions and the same spot arrangement, their compression behaviors and degrees differ. These differences can be directly attributed to the variation in the shape of the HC regions, as this is the only difference between the patch types. With 0.74 ± 0.04 normalized surface area, the star patch type compresses the most of all types. The *circle* patch only maximally compressed to 0.80 ± 0.04 of its initial surface area, and thus, the spot structure of the pattern impacts the maximum compression. The cause of a greater compression of the *star* patches might be the thinner HC regions of this type compared to the compacter regions within the *circle* type, despite the same proportion of HC regions with 49% of the total patch surface area (compare Table S1). Both types of patches showed the reverse compression regime for individual patches. However, whereas all *star* patches showed this one regime, the *circle* patches partly compress according to the second regime with a plateau instead of reversed compression, as shown in **Figure S10**. Even if the compression regime varies for the *circle* patches, the individual compression curves are quite similar (compare Figure S10a). In contrast, the individual compression curves of all *star* patches significantly differ from each other (compare Figure S10b). The *star* patches vary in their maximum compression values and applied pressures but possess a uniform compression behavior during the first 500 mbar. Hence, the point of reversed compression seems to define the *star* compression curves and is shifted between the individual curves, which is characteristic for the *star* patch type and distinguishes this type from all other types. In conclusion, the structure of the HC regions of the patches affects the compression degree and behavior of the patches.

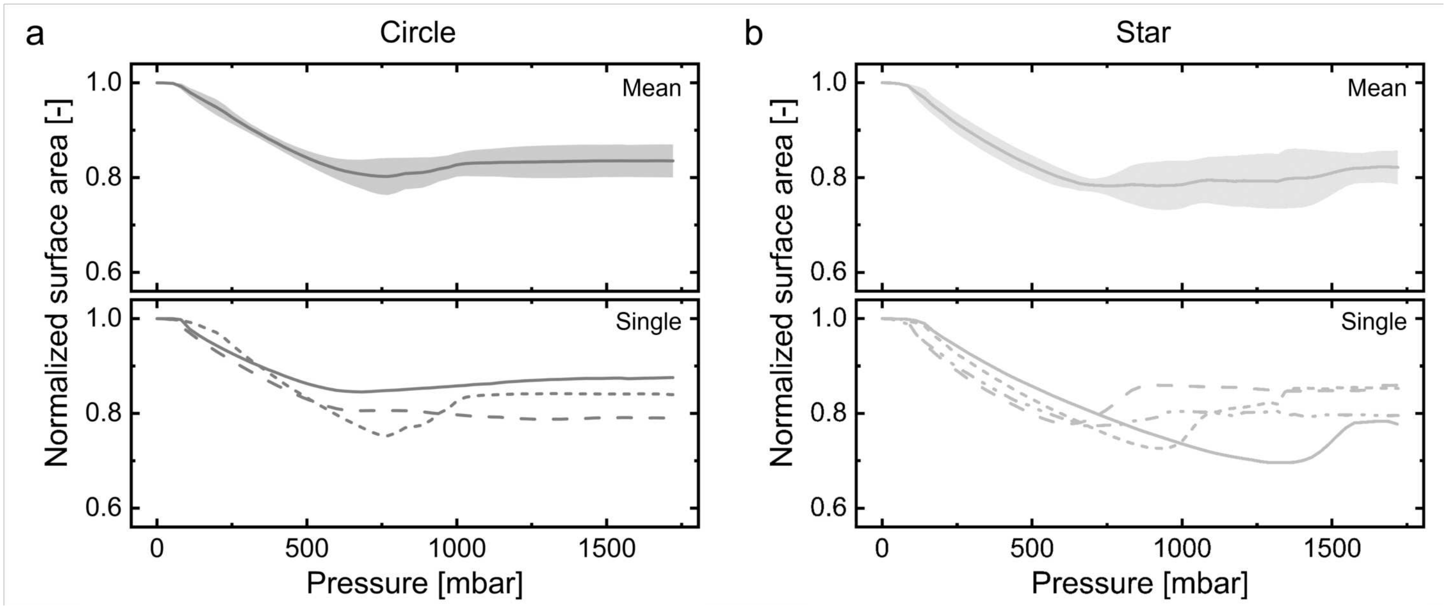

Figure S10: Single compression curves of all investigated (a) *circle* and (b) *star* patches and their respective means.

***Directionality* Patches Compared**

Within the *directionality* group, the *hor line* patch type exclusively shows reversed compression like the *star* type. However, the *hor line* patch type has a very reproducible compression with almost completely matching individual compression curves, resulting in a highly reproducible compression profile. The individual compression curves of the *hor line* and *ver line* patches are displayed in **Figure S11**. In opposition

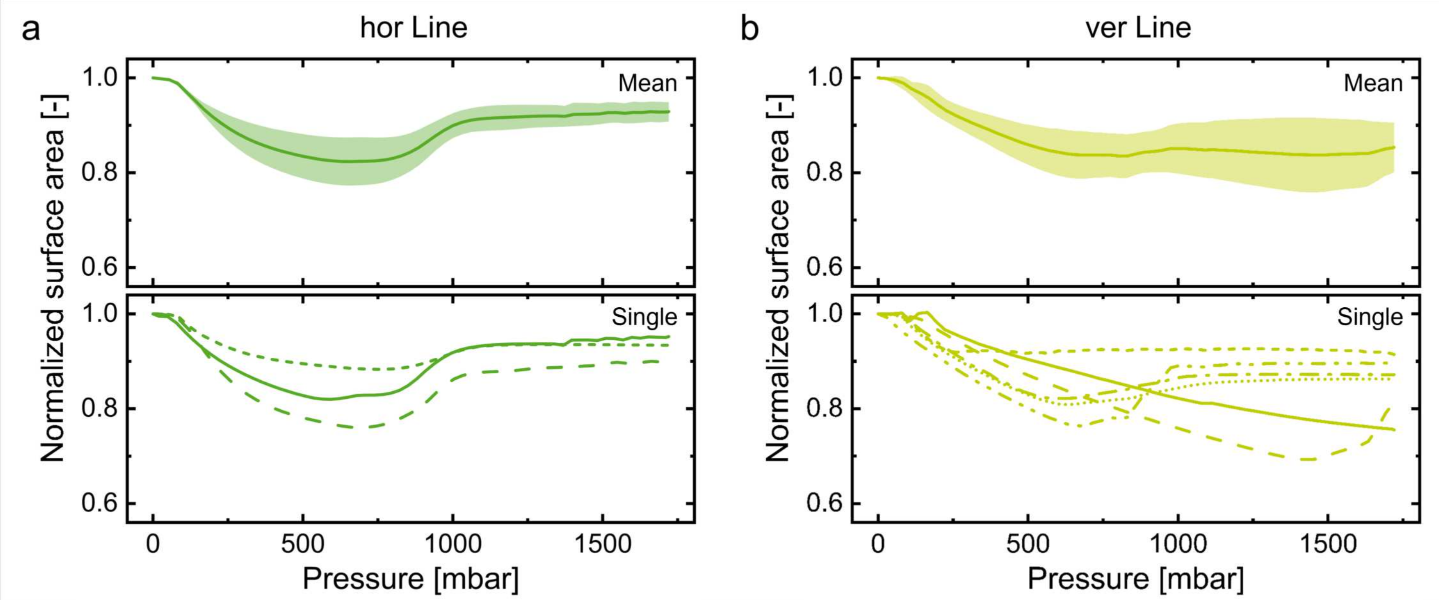

Figure S11: Single compression curves of all investigated (a) *hor line* and (b) *ver line* patches and their respective means.

to this behavior are the compressions of the two other *directionality* patch types *ver line* and *reference*. These patch types show all three compression regimes, dependent on the sample (compare Figure S11b for the *ver line* patch type). Furthermore, the mean maximum compression of both patches, *ver line* and *reference*, is identical with 0.79 ± 0.07. The *hor line* patch compressed slightly less with 0.82 ± 0.05. This difference between the patch types is still in the range of error bars. However, the average compression in the end state of the *hor line* type significantly differs from the other two patch types. Thus, this patch type shows an extremely reversed compressed surface area with $59 \pm 12\%$ as the highest of all patches. In

contrast to the *hor line* patch type, only $39 \pm 12\%$ of the maximum compression of the *ver line* patches and $48 \pm 7\%$ of the *reference* patches is reversed. Thereby, the patches of the *directionality* group all undergo a greater reversed compression than the ones of the *structure* group with around 30%, which might be caused by the large and continuously connected HC regions within the *directionality* patch types in at least one direction. In addition, the directionality of these regions seems to affect the degree of reversed compression. Thus, the directionality within the patch pattern provides a noticeable difference in their compression since the *hor line* and *ver line* patch types have identical patterns, which are only rotated by 90° but significantly differ in their compression. These differences are visible in the compression regime, the reproducibility, the maximum and end state compression, and especially in the reversed compression. Hence, by determining the directionality within the patch pattern, its compression behavior is significantly altered, while its structure and functionality remain, being a valuable tuning parameter for future applications. *Line* patch types, for example, can be used to provide flow channels or guide cell growth. Therefore, the directionality can be chosen freely without changing the functionality but by selecting the desired compression behavior. Furthermore, regarding the compression behavior, the proportion of the HC regions within the surface area of the patches is less important than the structure and directionality of these regions. The *structure* patch types both have an HC proportion of 49% and show diverse behaviors and strongly deviating compressions, as well as the *hor line* and the *ver line* types with 29% HC regions. Furthermore, the compression behavior of the *reference* type resembles more the *line* patches, even if it only consists of HC regions and is, therefore, more related to the *structure* patch types in this respect.

***Porosity* Patches Compared**

All *porosity* patches showed their maximum compression at pressures of around 40 mbar, which is significantly lower than the applied pressure profile of the other patch types of up to 1720 mbar (compare Figure 5d, main manuscript). Beyond 40 mbar, the *porosity* patches started to intrude the 5 µm large pores of the filter and thereby rupture at their LC regions. Hence, a higher pressure could not be applied to these patch types. Since the HC regions of the *porosity* patches are about 7 µm in diameter and hexagonally arranged circle shapes, they can be pressed through the filter in contrast to the *structure* or the *directionality* patches. The *w/o pore*, the *44 µm pore*, and the *95 µm pore* patch type maximally compressed to 0.83 ± 0.06, 0.80 ± 0.05, and 0.86 ± 0.03 of their surface area, respectively. Their maximum measured compressions are in the same order of magnitude as the ones of the other patch types, even though the applied pressure at the maximum compressions of the *porosity* group types is significantly lower. This comparison highlights the potential compression ability of the *porosity* patches due to their high flexibility and small and few HC regions. All *porosity* patches show very similar compression behaviors not only regarding their maximum compressions and pressure rates but also regarding their linear compression regime. This similarity is attributed to the patches' identical patch pattern in wide areas, only being distinguished by the introduced regions of a third level of porosity for the *44 µm pore* and the *95 µm pore* patch types. These regions, however, do not or only marginally alter the basic framework (shape, size, distance, and arrangement of the HC regions) of the patches, retaining their similarity to the *w/o pore* patch. The *44 µm pore* patch is more compressed than the other two patches of the *porosity* group but in the range of the error bars. To fabricate the *44 µm pore* patch, every second irradiation spot in every second row of spots on the patch's mask was omitted. This results in exchanging HC regions against regions of the third and most softest level of porosity. Hence, the whole framework gets more flexible, possibly revealing in an enhanced compression of the *44 µm pore* patch compared to the other *porosity* patches. Increasing the porosity of a patch by introducing a third level of porosity results in slightly different mechanical characteristics of the investigated patches. The achieved result depends on the distribution of the third level of porosity within the patch. If the basic framework is retained by introducing only distinct regions, like for the *95 µm pore* patch, the compression behavior of the patch area does not change, but the space the patch occupies decreases in the compressed state. If the additional level of porosity affects the distance respectively arrangement of the HC regions of the patch, like for the *44 µm pore* patch, an increased compression ability is achieved. More drastic porosity deviations might increase the extent of this effect. For both kinds of

alterations, the compression regime is not affected.

S6 Squeezing of Patches

The squeezing experiments of the patches enable the investigation of their folding capability. **Figure S12** shows the schematic setup of these experiments and the geometry of the microfluidic channel with a central constriction through which the patches were squeezed.

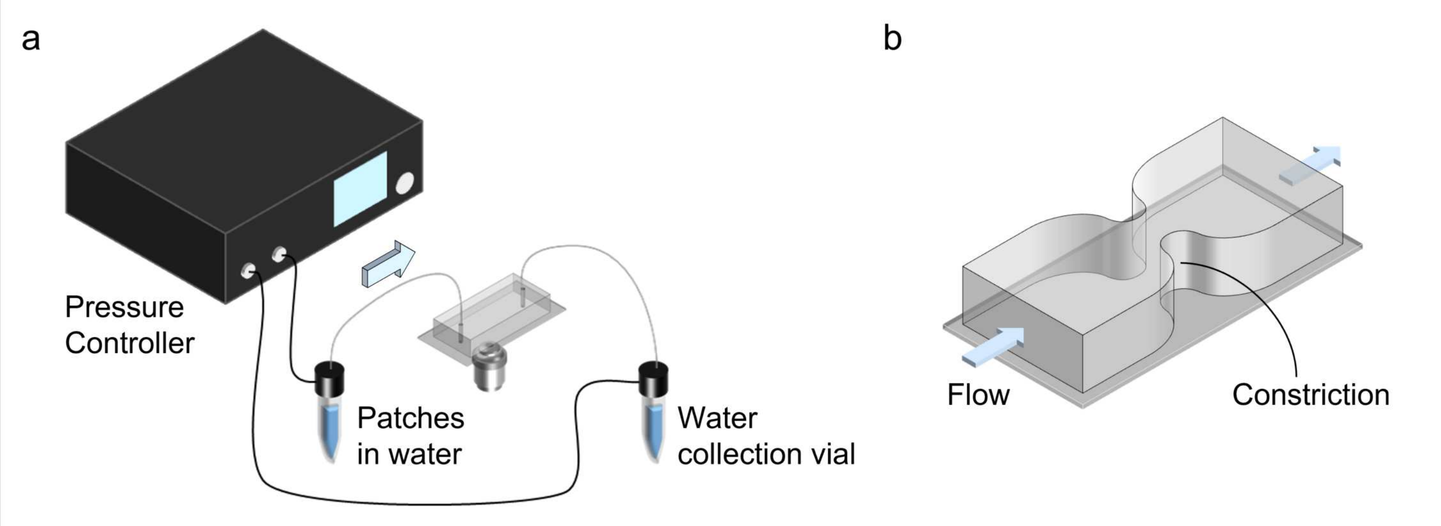

Figure S12: Schematic (a) setup to study the squeezing of patches and (b) detailed microfluidic channel scheme with displayed constriction.

S6.1 Visual Squeezing Investigation

Figures S13, S14, and S15 show the intrusion, half-through, and breakthrough state of the patches during squeezing.

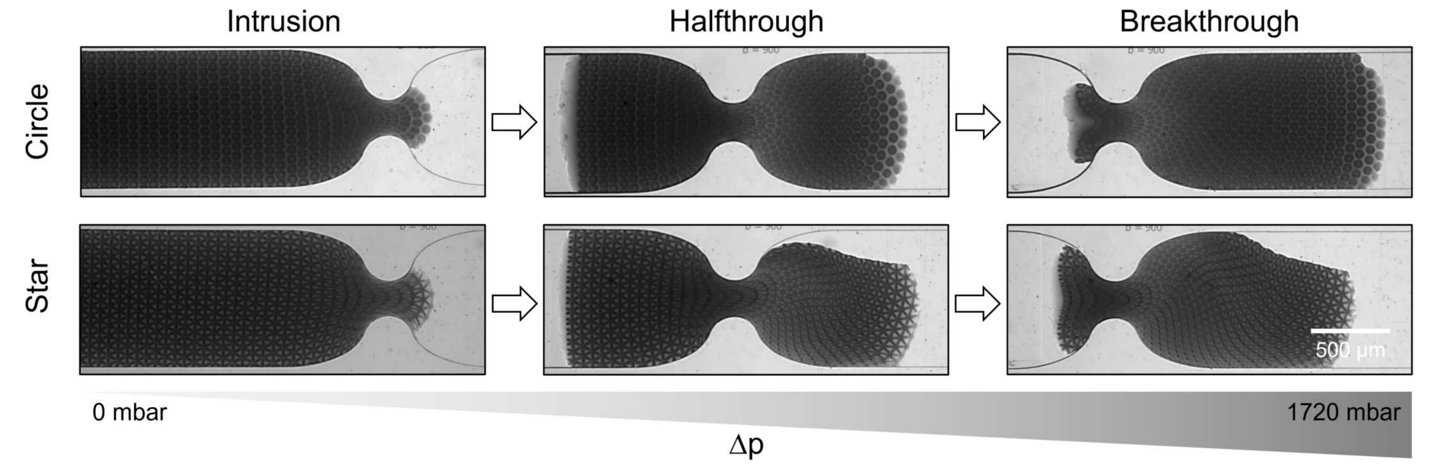

Figure S13: Squeezing states of the *structure* patch types *circle* and *star*. Scale bar (500 µm) applies to all images.

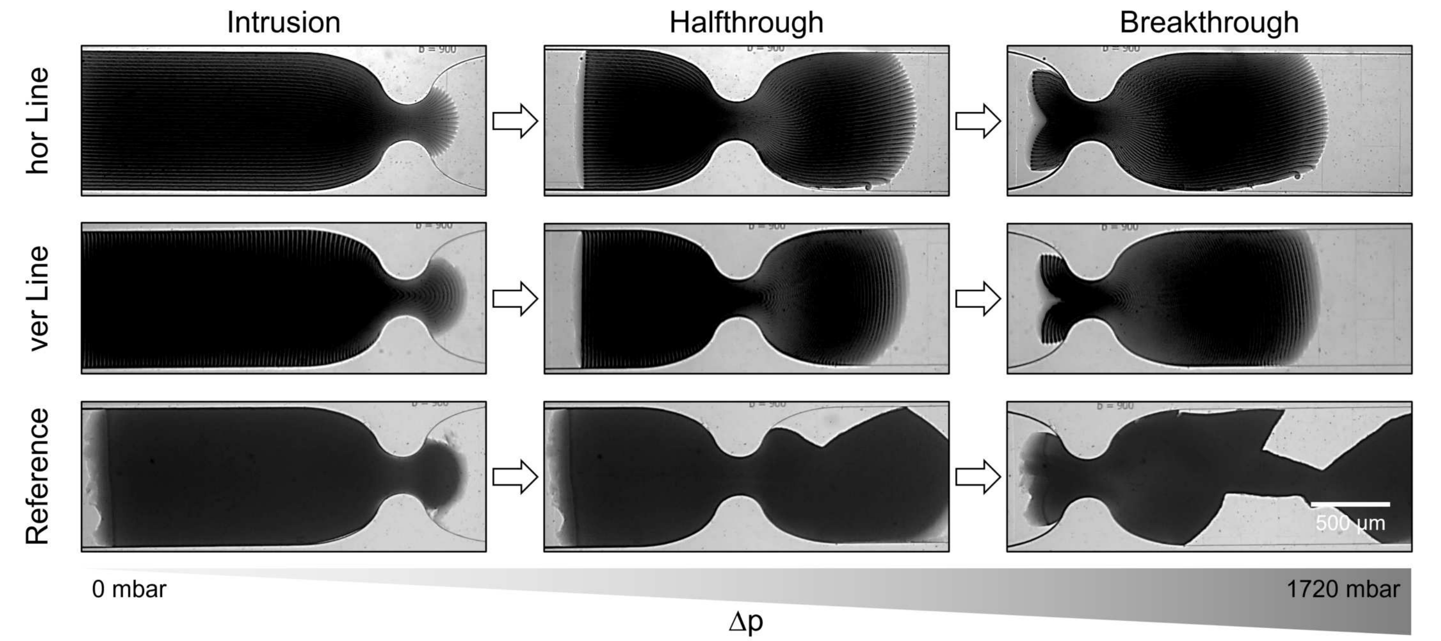

Figure S14: Squeezing states of the *directionality* patch types *hor line*, *ver line*, and *reference*. Scale bar (500 µm) applies to all images.

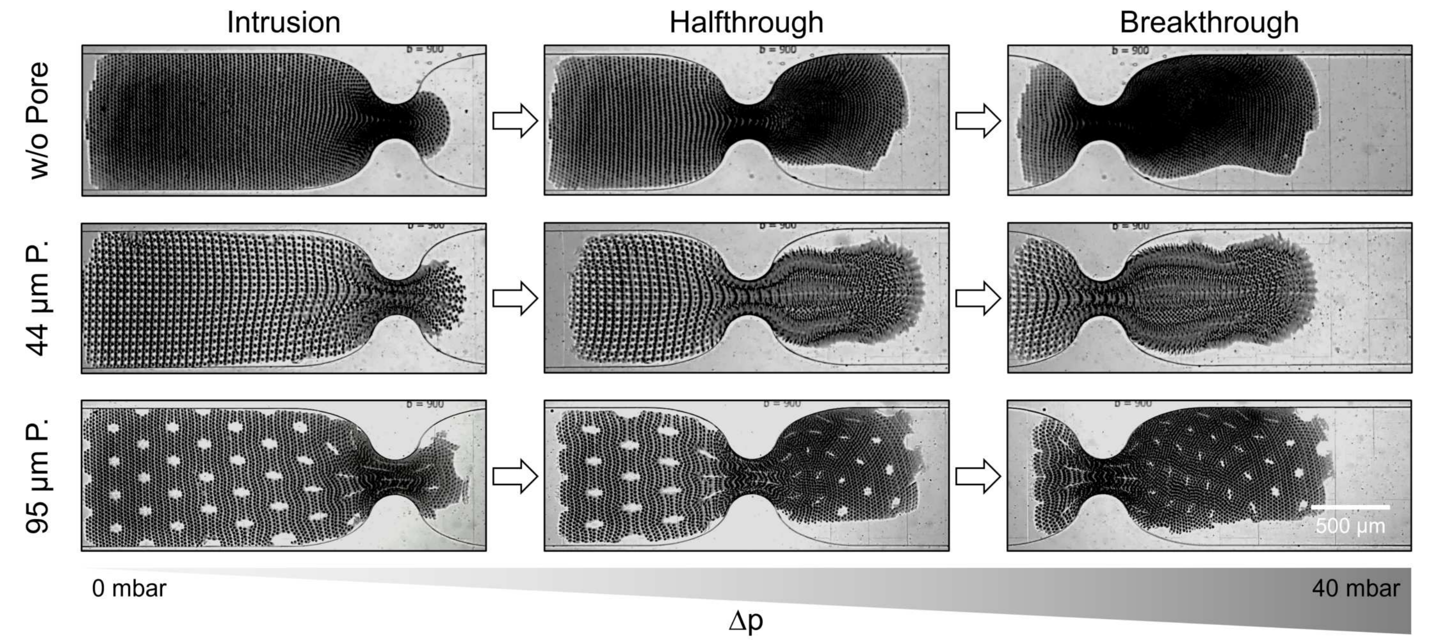

Figure S15: Squeezing states of the *porosity* patch types *w/o pore*, *44 µm pore*, and *95 µm pore*. Scale bar (500 µm) applies to all images.

Groove during Squeezing

During the squeezing of both *line* patch types, *hor line* and *ver line*, a characteristic deformation forms at the end of the patches right before the breakthrough, which is depicted in Figure S14. Thereby, a groove forms at the height of the constriction in the y-direction. This deformation is caused by how the remaining patch area passes the constriction and is accompanied by a strong parabolic forward protrusion when passing through the constriction. This deformation was observed for the *circle* type as well, and to some extent also for the *star* and *reference* type. The assumption is, therefore, that this deformation occurs in particular due to the low flexibility of the patches caused by their large HC regions.

S6.2 Squeezing Values

Table S3 shows time and pressure at the half-through and the breakthrough states of the patches during squeezing. Furthermore, the relative time and pressure between these two states are listed. These relative values arise from dividing the half-through through the breakthrough value each. These relative values as well as the half-through pressure are additionally depicted in **Figure S16**.

Table S3: Squeezing values.

	Half-through		Breakthrough		Half- vs. Breakthrough	
Patch type [-]	Time [s]	Pressure [mbar]	Time [s]	Pressure [mbar]	Relative time [-]	Relative pressure [-]
Circle	57 ± 2	1690 ± 25	90 ± 18	1719 ± 2	0.67 ± 0.15	0.98 ± 0.01
Star	31 ± 2	899 ± 49	35 ± 2	1001 ± 47	0.89 ± 0.01	0.90 ± 0.02
hor Line	56 ± 5	1605 ± 143	59 ± 2	1682 ± 55	0.95 ± 0.06	0.95 ± 0.06
ver Line	46 ± 4	1302 ± 110	54 ± 5	1531 ± 137	0.85 ± 0.06	0.85 ± 0.05
Reference	60 ± 0	1716 ± 0	68 ± 0	1717 ± 0	0.88 ± 0.00	1.00 ± 0.00
w/o Pore	50 ± 11	8.6 ± 1.2	56 ± 12	8.4 ± 1.2	0.89 ± 0.01	1.02 ± 0.06
44 µm Pore	27 ± 9	4.4 ± 1.6	30 ± 10	4.6 ± 1.9	0.90 ± 0.02	0.97 ± 0.09
95 µm Pore	30 ± 10	8.6 ± 1.1	32 ± 10	9.3 ± 1.3	0.94 ± 0.02	0.93 ± 0.04

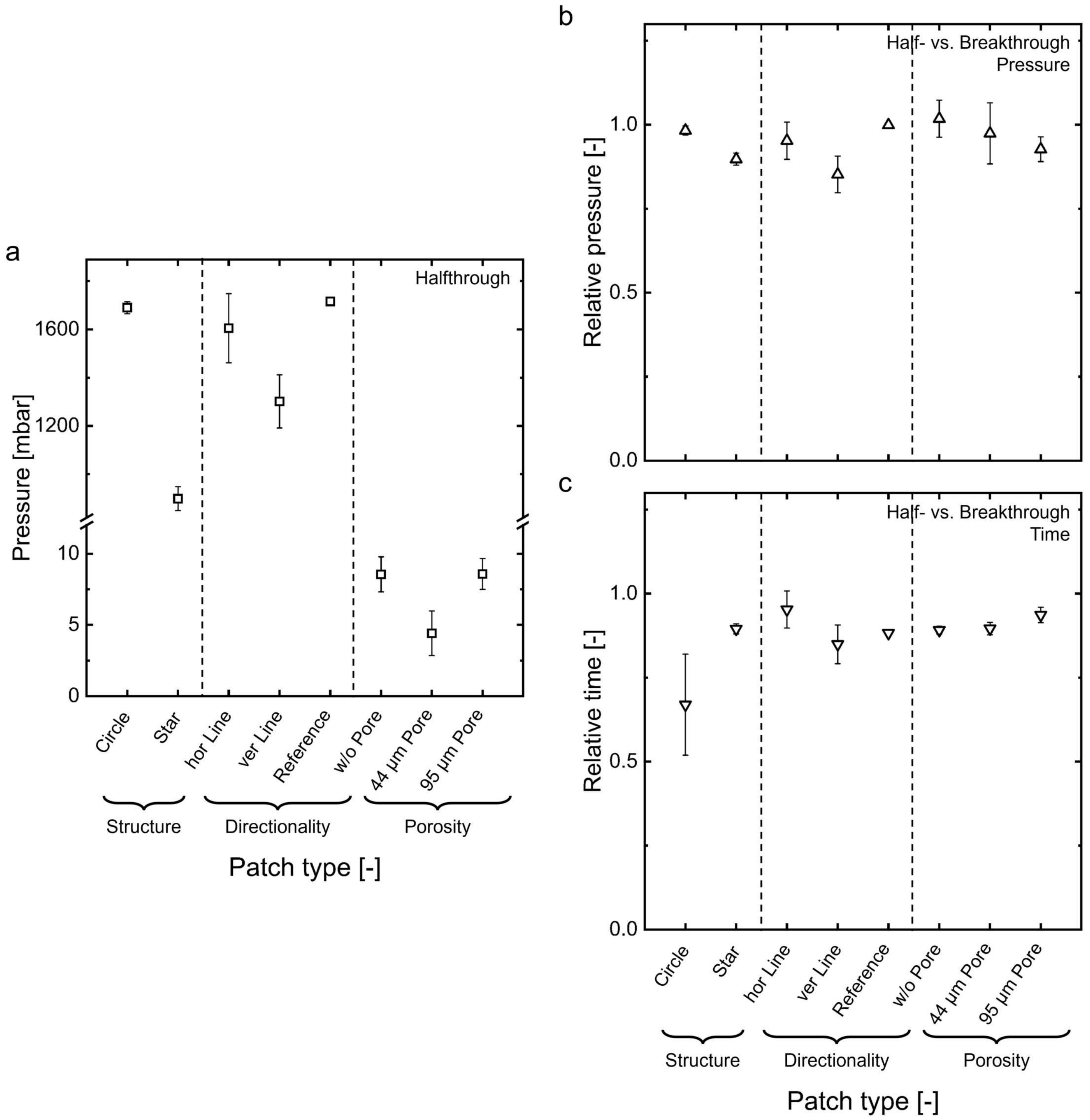

Figure S16: Squeezing values of two *structure*, all *directionality*, and all *porosity* patch types, showing (a) the half-through pressure and (b) the relative pressure and the (c) the relative time between the half-through and breakthrough state.

S6.3 Squeezing Behavior

The ability of patches to squeeze through constrictions is influenced by the structure, arrangement, and directionality of HC regions. In the *structure* and *directionality* groups, breakthrough pressure variations highlight the importance of both the HC region structure and pattern directionality. In the *porosity* group, flexibility is more closely related to the proportion and arrangement of HC regions.

***Structure* Patches Compared**

Within the *structure* patch group, there are great differences in breakthrough pressure. The *star* patch type squeezed through the constriction at a significantly lower average pressure of 1001 ± 47 mbar than the *circle* patch type with 1719 ± 2 mbar, even though both patch types comprise the same proportion of HC regions. This result matches the findings from the compression experiments. The structure of the HC regions as the only difference between the patch patterns enables facilitated squeezing for the *star* patch type, most likely due to the thinner and more distinct HC regions within its pattern. Hence, the experiments highlight the relevance of the structure of the HC regions for the patches' squeezing ability.

***Directionality* Patches Compared**

The *ver line* type breaks through the constriction at a lower average pressure of 1531 ± 137 mbar than the other two patch types of the *directionality* group. These have their breakthrough at 1682 ± 55 mbar for the *hor line* patch type and at 1717 mbar for the *reference* type. The breakthrough pressure of the *reference* type is only based on a measurement of a single patch. The additionally investigated four *reference* patches, all either ruptured or stuck in the constriction instead of fully surpassing the constriction. The one *reference* patch that surpassed the constriction ruptured as well behind the constriction, which is visible in Figure S14. Thus, the *reference* patch type shows the strongest resistance of all types regarding breakthrough since the patch only consists of one HC region without LC regions. Therefore, local compression by squeezing this patch type is only partially possible as the hydrogel reaches its compression limit. Thus, the proportion of the HC regions is relevant for the local compression and folding ability. However, this aspect is not the only relevant one. The *hor line* and *ver line* patches differ in their breakthrough pressure and squeezing behavior, even though they have an identical HC proportion, which also applies to the comparison of the *structure* patches. Furthermore, the *star* type surpassed the constriction at a significantly lower pressure than the *hor line* and *ver line* types, although the *star* types proportion of HC regions is with 49% significantly higher than the 29% proportion of the *line* patch types. Thus, the structure of the HC regions and the directionality within the pattern of the patches also impact their local compression and folding ability.

***Porosity* Patches Compared**

The patches of the *porosity* group squeeze and break through the constriction at considerably lower pressure values of 5-9 mbar than all other patch types at minimum 1000 mbar. The small size of the HC regions and their low proportion of 7% or less within the *porosity* patch types explain this pressure difference during breakthrough. The great amount of a flexible and interconnected LC region, as well as the hexagonal scattered pattern, result in particularly high flexibility of the *porosity* patches (compare Figure S4). When comparing the *porosity* patch types with each other, the squeezing behavior of the *w/o pore* and the *95 µm pore* patch type resembles, which is caused by the identical framework of both patch types. Both patch types have a proportion of 7% of HC regions within the patch area and break through the constriction at 8 ± 1 mbar and 9 ± 1 mbar, respectively. In contrast, the *44 µm pore* patch type has a breakthrough pressure of 5 ± 2 mbar. The most reasonable explanation for this variance compared to the other two *porosity* patch types is the lower proportion of HC regions of 4% for the *44 µm pore* patch. Due to this decrease in proportion, the *44 µm pore* patch type has increased flexibility through the increased amount

of LC regions as folding points. These additional regions are even lower crosslinked due to the greater distance between HC regions. However, the *95* µm *patch* has holes, which should increase the flexibility as well, but which had no remarkable impact on the squeezing behavior. Hence, the important difference might be the change in the framework of the *44 µm pore* type compared to the other *porosity* types, namely the change of the distance respectively arrangement of the HC regions. By regularly missing single HC regions, the whole patch seems to get more flexible. Thus, by adapting the framework, the flexibility of the patches can be tuned, whereas scattered holes have less impact on the local compression and folding ability.

S7 Cell Cultivation

Table S4: Cell cultivation values.

Patch material [-]	Patch type [-]	Cell area [µm^2]	Eff. Young's modulus [kPa]
PEGDA	Circle 7 µm	331 ± 144	1.0 ± 0.6
	Circle 52 µm	717 ± 263	-
	Reference	1173 ± 271	30.2 ± 1.8
	No patch	1987 ± 860	-
PNIPAM	Circle 7 µm	916 ± 248	-
	Circle 52 µm	1046 ± 597	8.4 ± 1.3
	Reference	1116 ± 415	14.6 ± 0.6
	No patch	1637 ± 488	-

Effective Young's Modulus of Patches

Effective Young's moduli of the patches used for cell cultivation are displayed in Figure 7d, main manuscript. Young's moduli of the *circle 7 µm* PNIPAM patch and of the *circle 52 µm* PEGDA patch could not be detected via nanoindentation. The *circle 7 µm* PNIPAM patch seemed to create too little resistance to the probe to be measured, whereas the *circle 52 µm* PEGDA patch seemed to interact with the probe, generating no reasonable or analyzable data.
